# Circulating fatty acid binding protein 4 (FABP‐4) concentrations and mortality in individuals with colorectal cancer in the European Prospective Investigation into Cancer and Nutrition study

**DOI:** 10.1002/ijc.70090

**Published:** 2025-08-26

**Authors:** Thu Thi Pham, Katharina Nimptsch, Krasimira Aleksandrova, Mazda Jenab, Veronika Fedirko, Anja Olsen, Anne Tjønneland, Claire Cadeau, Gianluca Severi, Matthias B. Schulze, Renée Turzanski Fortner, Verena Katzke, Claudia Agnoli, Carlotta Sacerdote, Rosario Tumino, Simona Signoriello, Camino Trobajo‐Sanmartín, Jesús‐Humberto Gómez, María‐Dolores Chirlaque, Maria‐Jose Sánchez, Marta Crous‐Bou, Anne May, Alicia Heath, Dagfinn Aune, Elisabete Weiderpass, Tobias Pischon

**Affiliations:** ^1^ Molecular Epidemiology Research Group Max‐Delbrueck‐Center for Molecular Medicine in the Helmholtz Association (MDC) Berlin Germany; ^2^ Department of Epidemiological Methods and Etiological Research Leibniz Institute for Prevention Research and Epidemiology—BIPS Bremen Germany; ^3^ Faculty of Human and Health Sciences University of Bremen Bremen Germany; ^4^ Nutrition and Metabolism Branch, International Agency for Research on Cancer (IARC‐WHO), World Health Organization Lyon France; ^5^ Department of Epidemiology University of Texas M.D. Anderson Cancer Center Houston Texas USA; ^6^ Department of Epidemiology Rollins School of Public Health, Emory University Atlanta Georgia USA; ^7^ Danish Cancer Society Research Center Copenhagen Denmark; ^8^ Department of Public Health University of Århus Århus Denmark; ^9^ Department of Public Health University of Copenhagen Copenhagen Denmark; ^10^ Paris‐Saclay University, UVSQ, Inserm, Gustave Roussy, CESP Villejuif France; ^11^ Department of Statistics, Computer Science and Applications “G. Parenti” University of Florence Florence Italy; ^12^ Department of Molecular Epidemiology German Institute of Human Nutrition Potsdam‐Rehbruecke Nuthetal Germany; ^13^ Institute of Nutritional Science, University of Potsdam Nuthetal Germany; ^14^ Division of Cancer Epidemiology German Cancer Research Center (DKFZ) Heidelberg Germany; ^15^ Department of Research Cancer Registry of Norway, Norwegian Institute of Public Health Oslo Norway; ^16^ Epidemiology and Prevention Unit, Department of Epidemiology and Data Science Fondazione IRCCS Istituto Nazionale dei Tumori Milan Italy; ^17^ Department of Health Sciences University of Eastern Piedmont Novara Italy; ^18^ Unit of Epidemiology Local Health Unit of Novara Novara Italy; ^19^ Hyblean Association for Epidemiology Research, AIRE ONLUS Ragusa Italy; ^20^ Medical Statistics Units University of Campania “Luigi Vanvitelli” Caserta Italy; ^21^ Instituto de Salud Pública y Laboral de Navarra Pamplona Spain; ^22^ Centro de Investigación Biomédica en Red de Epidemiología y Salud Pública (CIBERESP) Madrid Spain; ^23^ Navarre Institute for Health Research (IdiSNA) Pamplona Spain; ^24^ Department of Epidemiology Regional Health Council, IMIB‐Arrixaca Murcia Spain; ^25^ Department of Epidemiology Regional Health Council, IMIB‐Arrixaca, Murcia University Murcia Spain; ^26^ Escuela Andaluza de Salud Pública (EASP) Granada Spain; ^27^ Instituto de Investigación Biosanitaria ibs.GRANADA Granada Spain; ^28^ Unit of Nutrition and Cancer Cancer Epidemiology Research Program, Catalan Institute of Oncology (ICO) Catalonia Spain; ^29^ Bellvitge Biomedical Research Institute (IDIBELL), L'Hospitalet de Llobregat Barcelona Spain; ^30^ Department of Epidemiology Harvard T.H. Chan School of Public Health Boston Massachusetts USA; ^31^ Julius Center for Health Sciences and Primary Care, Epidemiology & Health Economics University Medical Center Utrecht Utrecht The Netherlands; ^32^ Department of Epidemiology and Biostatistics School of Public Health, Imperial College London London UK; ^33^ Department of Nutrition Oslo New University College Oslo Norway; ^34^ Department of Research, Cancer Registry of Norway Norwegian Institute of Public Health Oslo Norway; ^35^ International Agency for Research on Cancer (IARC) World Health Organization Lyon France; ^36^ Charité‐Universitätsmedizin Berlin Corporate Member of Freie Universität Berlin and Hum‐boldt‐Universität zu Berlin Berlin Germany; ^37^ Biobank Technology Platform Max‐Delbrueck‐Center for Molecular Medicine in the Helmholtz Association (MDC) Berlin Germany

**Keywords:** EPIC, FABP‐4, human fatty acid binding protein‐4, incident colorectal cancer, mortality

## Abstract

Human fatty acid binding protein‐4 (FABP‐4), a protein elevated in obesity that promotes colon cancer cell invasiveness and metastasis, may be associated with higher mortality in individuals with colorectal cancer (CRC) and may serve as a mediator of the obesity–mortality association in these individuals. We used a causal diagram to inform covariate selection and applied Cox proportional hazards models to estimate hazard ratios (HRs) for CRC‐specific, non‐CRC‐specific, and all‐cause mortality by FABP‐4 levels measured in baseline blood samples from 1371 incident CRC cases from the European Prospective Investigation into Cancer and Nutrition cohort. Competing risk analyses were adapted for CRC and non‐CRC deaths. Mediation analyses were conducted to estimate total effects (TEs), direct effects (DEs), and mediation proportions (MPs) by FABP‐4 of pre‐diagnostic body mass index (BMI) on mortality. In the fully adjusted model including BMI, higher circulating FABP‐4 concentrations were associated with higher CRC mortality (HR_Q4vsQ1_ = 1.49; 95% CI: 1.11–2.00) and all‐cause mortality (HR_Q4vsQ1_ = 1.49; 95% CI: 1.15–1.93), but not statistically associated with non‐CRC mortality (HR_Q4vsQ1_ = 1.51; 95% CI: 0.82–2.76). The TE and DE per 5 kg/m^2^ of BMI on all‐cause mortality were 1.21; 95% CI: 1.10–1.34, and 1.13; 95% CI: 1.02–1.26, respectively, with a MP of 34.5% (*p* = .002) by FABP‐4. For CRC‐specific and non‐CRC‐specific mortality, MPs by FABP‐4 were 33.7% (*p* = .03) and 36.1% (*p* = .02), respectively. In conclusion, higher concentrations of FABP‐4 were associated with higher CRC‐specific and all‐cause mortality in individuals with CRC. FABP‐4 was a significant partial mediator of the adiposity‐mortality relationship in individuals with CRC.

## Graphical abstract

What's new?

Higher pre‐diagnostic body mass index has been associated with higher risk of mortality in individuals with colorectal cancer; however, the mechanism remains unclear. This prospective analysis of incident colorectal cancer cases within the European Prospective Investigation into Cancer and Nutrition cohort study addressed potential collider stratification bias and suggests that higher pre‐diagnostic FABP‐4 concentrations may be associated with higher colorectal cancer‐specific and all‐cause mortality. Moreover, FABP‐4, a protein involved in metabolic regulation and inflammation that has been found to be elevated in obesity, could partially explain the body mass index–mortality association in individuals with colorectal cancer.

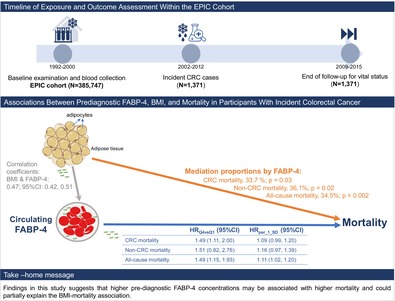

**Abbreviations**
A‐FABP: adipocyte‐FABP
aP2: adipocyte protein 2
BMI: body mass index
CIFs: cumulative incidence functions
CRC: colorectal cancer
CRP: C‐reactive protein
CVDs: cardiovascular diseases
DE: direct effects
ELISA: enzyme‐linked immunosorbent assay
EPIC: the European Prospective Investigation into Cancer and Nutrition
*ɛ*(WC|BMI + height): the residual values of WC when regressed on BMI and height
FABP‐4: Fatty acid binding protein‐4
FFQ: food frequency questionnaire
FRAP: ferric reducing ability of plasma
HDLC: high‐density lipoprotein cholesterol
IARC: International Agency for Research on Cancer
ICD‐10: the 10th revision of the International Classification of Diseases, Injuries, and Causes of Death
IE: indirect effect
IGFBP: insulin‐like growth factor‐binding protein
IQR: interquartile range
MP: mediation proportion
QC: quality controls
ROM: reactive oxygen metabolites
SEER: Surveillance, Epidemiology, and End Results
TE: total effect
WC: waist circumference

## 1 INTRODUCTION

Obesity (defined as body mass index [BMI] ≥ 30 kg/m^2^) is “a condition of abnormal or excessive fat accumulation in adipose tissue that can compromise health,” as described by the World Health Organization.^1^ Obesity is a well‐established risk factor for colorectal cancer (CRC),^2^ and may also play a role in survival after CRC diagnosis.^3^, ^4^ Thus, a study using data from the European Prospective Investigation into Cancer and Nutrition (EPIC) study among persons with CRC showed that every 5 kg/m^2^ higher pre‐diagnostic BMI was associated with a 10% higher risk of CRC‐specific death and a 12% higher risk of all‐cause death.^5^ However, the mechanism underlying this association is unclear.^5^, ^6^ It was suggested that research on obesity‐related biomarkers should be conducted to provide further evidence.^7^, ^8^

Fatty acid binding protein‐4 (FABP‐4), also known as adipocyte protein 2 (aP2) or adipocyte‐FABP (A‐FABP), is a 132 amino acid protein in humans mainly expressed in mature adipocytes, macrophages, and endothelial cells that functions as a lipid carrier.^9^, ^10^ FABP‐4 is a versatile protein with both intracellular and extracellular forms.^11^ The intracellular form (identified first) has antioxidant activity and multiple functions, including the regulation of fatty acid uptake and lipogenesis, and the facilitation of intracellular fatty acid trafficking.^11^ The concentration of the circulating extracellular form depends on fasting status, the degree of lipolysis, and appears to be regulated by obesity‐related signals.^11^ Circulating FABP‐4 is involved in lipid modulation, metabolic regulation (including insulin resistance), and inflammation.^11^ Increased circulating concentrations of FABP‐4 were observed in individuals with obesity as compared to those with normal‐weight,^12^, ^13^, ^14^ and a Mendelian Randomization (MR) study found a strong positive association between BMI and circulating FABP‐4 concentrations.^15^ We previously found no strong support for a causal role of circulating FABP‐4 in the development of CRC in an investigation that included both an individual‐level prospective cohort and an MR analysis.^14^ However, some experimental studies in normal human endothelial cells^16^ and colon cancer cells^17^ suggested that FABP‐4 may be involved in promoting angiogenesis and tumor growth.^16^, ^17^ Therefore, FABP‐4 may be relevant for the prognosis of CRC patients and could qualify as a potential biomarker linking obesity and risk of mortality in individuals who are diagnosed with CRC. However, no study has been implemented to investigate these relationships so far. Therefore, we aimed to investigate the associations between pre‐diagnostic circulating FABP‐4 and CRC‐specific mortality, non‐CRC‐specific mortality, and all‐cause mortality in participants with incident CRC in the EPIC cohort. We further performed causal mediation analyses to estimate the mediation proportions (MPs) of FABP‐4 for the association between BMI and risk of mortality in individuals with CRC.

## 2 MATERIALS AND METHODS

### 2.1 The European Prospective Investigation into Cancer and Nutrition (EPIC) cohort

A prospective study was designed within the EPIC cohort by including only incident CRC cases. Details of the EPIC study have been published elsewhere.^18^ In brief, the EPIC study, initiated by the International Agency for Research on Cancer (IARC) in 1990, is one of the largest cohort studies in Europe, with continued follow‐up of study participants in 25 centers in 10 western European countries. A total of 519,978 participants were recruited between 1992 and 2000, representing a population aged 35–70 years.^18^ The baseline data include (i) anthropometry with either at‐study‐center measured or self‐reported values of weight, height, hip circumference, and waist circumference (WC); (ii) information from lifestyle and health standard questionnaires including reproductive history, physical activity, tobacco smoking, alcohol consumption, occupational history, socio‐economic status, and previous illnesses; (iii) dietary data from (semi) quantitative food frequency questionnaires (FFQs), including a wide range of food items and individual average standard portions. Anthropometry measurement has been described in detail previously.^5^ BMI was calculated as weight in kilograms divided by height in meters squared (kg/m^2^), with weight and height measured barefoot.^5^ WC was measured at the narrowest point or midway between the ribs and the iliac crest.^5^ Measurements were adjusted for clothing to minimize variability between centers.^5^ In addition, blood samples were collected from 385,747 participants at the time of recruitment and aliquoted into blood plasma, blood serum, white blood cells, and red blood cells.^18^ Samples were stored in the vapor phase of liquid nitrogen at −196°C (in plastic straws for most centers at IARC central biorepository and in tubes for the Danish centers locally), or in freezers at −70°C (in tubes for the Swedish centers locally).

Cancer incidence was identified by two methods: record linkage to regional cancer registries (Denmark, Italy, the Netherlands, Norway, Spain, Sweden, and the United Kingdom) or an active follow‐up by various means, including health insurance data, pathology data, and data on liaising with study participants and their closest living relatives (France, Germany, Naples, and Greece). Vital status was also followed up through either record linkage to regional/national mortality registries or active follow‐up (France, Germany, and Greece). Both cancer incidence and mortality were coded in accordance with the 10th revision of the International Classification of Diseases, Injuries, and Causes of Death (ICD‐10).

### 2.2 Design of the present study: CRC cases ascertained and followed up for mortality

Participants in this study were incident CRC cases from the EPIC study, identified during follow‐up from baseline to the closure date of the index centers, which ranged from September 2002 to December 2012. The CRC cases included participants with colon cancer (ICD‐10 code: C18.0–C18.7), rectal cancer (C19–C20), and cancer in the overlapping or unspecified areas within the colon (C18.8 and C18.9). The center‐specific dates of follow‐up cessation for vital status of these CRC cases ranged from December 2009 to January 2015. Due to administrative issues, data from Greece and Norway were not included in the present study; however, the two countries contributed only a small portion of CRC cases (6.4%),^19^ which were not expected to influence the estimates in this study. In total, 1371 CRC cases (807 colon, 496 rectal, and 68 unspecified colon cancer) with bio‐samples available were included in the present study.

### 2.3 Laboratory analysis of FABP‐4

FABP‐4 was measured in baseline blood serum samples by enzyme‐linked immunosorbent assay (ELISA) by BioVendor (BioVendor Laboratory Medicine, Inc.; Brno, Czech Republic). Inter‐assay coefficients of variation during the laboratory analysis were under 6.5% for all quality controls (QC). The means of intra‐assay coefficients of variation for high QC and low QC were 2.49% and 3.58%, respectively.

### 2.4 Final dataset and variables

Missing baseline data were encountered in WC [75 (5.5%)], smoking status [11 (0.8%)], physical activity index [87 (6.3%)], education level [44 (3.2%)], and all dietary variables [4 (0.3%)]. In addition, we defined participants as having diabetes if they had HbA1c ≥6.5 or self‐reported diabetes; data for this variable were missing in 68 (5.0%) CRC cases.

Prognostic factors, such as age at diagnosis, tumor subsite, and tumor stage, were retrieved from patients' medical records.^20^ There were three systems used to classify stage data among EPIC study centers, including TNM staging, Dukes classification, or the categories “localized/metastatic/metastatic regional/metastatic distant.” Therefore, a new variable for tumor stages (I, II, III, and IV) was previously created during the harmonization process to cover a broad category of tumor stages.^20^ However, even after this process, data on tumor stage were missing in 185 (13.5%) cases.

### 2.5 Statistical analysis

We categorized FABP‐4 into quartiles based on the sex‐specific distribution of FABP‐4 concentrations in a cohort of non‐cancer controls in the EPIC data that have been reported previously.^14^ The cut‐off points (in ng/mL) for the quartiles were, for men: ≤9.00; 9.01 to ≤12.10; 12.11 to ≤16.00; and >16.00; for women: ≤14.00; 14.01 to ≤18.30; 18.31 to ≤24.50; >24.50, respectively.^14^ Characteristics of the study population were summarized across sex‐specific quartiles. We further calculated age‐and‐sex partial Spearman correlation coefficients between FABP‐4 and markers of adiposity, inflammation, and metabolism.

The primary outcome was CRC‐specific mortality, while non‐CRC‐specific mortality and all‐cause mortality were the secondary outcomes. We employed competing risk survival analyses to estimate cause‐specific hazards or subdistribution hazards of death.^21^ For the main analyses, we used cause‐specific Cox proportional hazard models as proposed by Prentice et al.^22^ and censored ‘competing events’. In a sensitivity analysis, we also applied the Fine‐Gray proportional subdistribution hazard models,^23^ modeling hazards based on the cumulative incidence function. For all‐cause mortality, the conventional Cox proportional hazards models were used. All models were regressed with the underlying time variable as time from CRC diagnosis to the time of death or last follow‐up and performed by PROC PHREG in SAS. Censored observations were defined as CRC cases lost to follow‐up or were still alive at the end of administrative follow‐up (“end of study censoring”).^24^, ^25^

**FIGURE 1:**
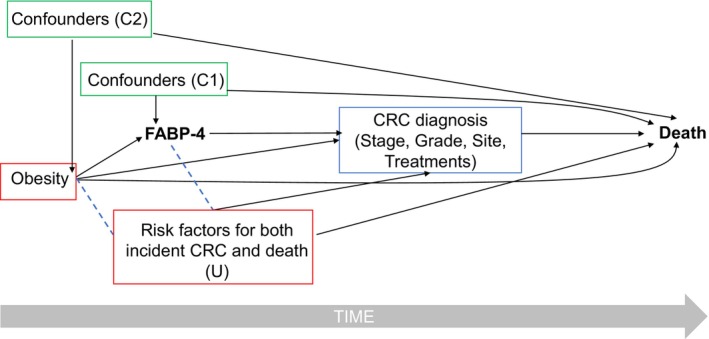
Causal diagram illustrating the relationship between FABP‐4, colorectal cancer (CRC), and death after CRC diagnosis. CRC diagnosis is a collider on the paths of U → CRC diagnosis ← FABP‐4. The blue box around “CRC diagnosis” indicates that analyses are conducted among individuals with incident CRC (or conditioning on CRC diagnosis). The analysis conditioning on CRC diagnosis may lead to “collider stratification bias.” This opens a non‐causal spurious association between FABP‐4 and (U) (dashed blue line). These opened paths can be blocked (red boxes) by adjusting for (U) and Obesity/BMI. The risk factors for incident CRC can be obtained from the expert report from the World Cancer Research Fund. Conventional confounders in this diagram were ancestors of both FABP‐4 levels and death (C1), and both obesity and death (C2). The paths through these conventional confounders were open and can be blocked (green boxes) by adjusting for C1 and C2.

Covariates were selected as (1) factors that could potentially act as a confounding factor in the relationship between FABP‐4 and mortality, and (2) factors (U) that could link to FABP‐4 (the exposure) by backdoor paths (e.g., U → CRC diagnosis ← FABP‐4), see Figure 1 and related discussion in the work of Osadnik et al.^26^ The latter set likely represented risk factors of incident CRC as reported by the World Cancer Research Fund expert,^27^ and was selected to address concerns about the potential collider stratification bias when conditioning on CRC diagnosis (directed acyclic graphs, Figure 1).^28^, ^29^ Adjustments for treatments and prognostic factors were not considered appropriate as it could open backdoor paths. Adiposity measures, including BMI and WC, were considered as potential confounders and included in a separate model. Eventually, three models were employed in the analyses: Model 1 stratified by country and adjusted for sex (based on the box‐plot of FABP‐4 levels by country in Figure S1); Model 2 additionally adjusted for age at diagnosis (continuous), smoking status (never, past, current smoker), education (none, primary school, technical/professional school, secondary school, or longer education), sex‐specific categories of physical activity (inactive, moderately inactive, moderately active, active), polyps of the large bowel (yes, no), alcohol consumption per day (continuous), and daily intake of red meat, processed meat, fish and shellfish, calcium, dietary fiber, dairy products, vegetables, and fruits (all variables were continuous); and Model 3 added to Model 2 by additionally adjusting for BMI and the residual values of WC when regressed on BMI and height (*ɛ*(WC|BMI + height)).

We examined the patterns of missing data in each of the covariates, assumed missing at random, and imputed the missing data by multiple imputations using chained equations. Details of variables included in the multiple imputation are described in Data and Methods in Data S1. Twenty imputed datasets were generated using SAS PROC MI, and all parameters (e.g., estimates from Model 2 and Model 3) computed from imputed datasets were combined using PROC MIANALYZE.

The presence of nonlinearity was assessed by determining whether the addition of nonlinear terms—including cubic spline or polynomial terms—of FABP‐4 significantly improved the fit of Model 3 using a likelihood ratio test. The non‐significance of the tests suggested no evidence of a non‐linear relationship (Table S1). In addition, we flexibly modeled and graphically presented the relationship between FABP‐4 levels and each of the outcomes using restricted cubic splines with knots placed at 4 and 6 evenly spaced percentiles of FABP‐4 distributions (Figure S2). These linearity tests and plots supported the analyses of FABP‐4 modeled as a continuous variable in its original scale (ng/mL) (also used in cubic splines). We estimated hazard ratios (HRs) with respective 95% confidence intervals (CIs) continuously for one standard deviation (SD) in FABP‐4 levels as well as in categories for the 2nd, 3rd, and 4th compared to the 1st quartile of FABP‐4. The proportional hazards (PH) assumption was tested and we observed (i) no significant estimates of time‐dependent covariates which were generated as the interactions of a log function of time with the FABP‐4 variable (*p* = .14) and with BMI (*p* = .48); and (ii) no systematic patterns of scaled Schoenfeld residuals of FABP‐4 and BMI over follow‐up time (Figure S3).

Interactions were assessed across strata of pre‐defined potential effect modifiers, including sex (men, women), tumor site (colon, rectum), tumor stage (I, II, III, IV), time to CRC diagnosis (≤2, 2 to ≤8, and >8 years), baseline diabetes status (yes, no), baseline BMI (18.5≤ to <25, 25≤ to <30, and ≥30), by adding the interaction term of the index variables to the final model (Model 3). Subgroup analyses were performed for modifiers with interaction *p*‐values <.20. Missing data in tumor stage were replaced by multiple imputations before stratification. As sensitivity analyses, we re‐estimated the hazard risk by excluding an extreme observation of FABP‐4, by using a different competing risk analysis model (Fine–Gray proportional subdistribution hazard models), and by using a complete case analysis.

We further performed causal mediation (counterfactual approach) analyses to estimate the extent to which FABP‐4 mediates the relationship between BMI and mortality. First, a counterfactual‐based approach was used to estimate the total effects (TEs, i.e., the overall effect of BMI on CRC‐specific mortality) and the direct effects (DEs, i.e., the effect of BMI on CRC‐specific mortality when holding FABP‐4 constant) of BMI on mortality.^30^ Second, the MPs along with their 95% CIs were estimated using the *difference method*, defined MP = 1 − log(HR^DE^)/log(HR^TE^), enabled by a data duplication algorithm and a generalized estimation equations approach under the g‐linkability assumption.^30^ The causal mediation analyses were performed using a SAS macro %mediate, which was designed for time‐to‐event data with a stratified sample design.^31^ As a sensitivity analysis, we estimated again the DEs and indirect effects (IEs) under multivariate normality using the standard product‐of‐coefficients methods.^32^

We estimated that the minimum detectable hazard ratio (HR) per 1 ng/mL unit of FABP‐4 level is 1.015, or the HR per 1 SD of FABP‐4 is 1.125, given a sample size of 1371 participants, a standard deviation of FABP‐4 concentrations of 8.9 ng/mL,^14^ a CRC‐specific mortality rate of 0.33,^33^ a type I error probability of 5%, and a power of 80%. SAS Enterprise Guide, version 8.3.2 (SAS Institute, Inc., Cary, NC) was used for all statistical analyses. All tests were performed at a two‐tailed significance level of .05 for the determination of statistical significance.

## 3 RESULTS

The median time from baseline examination (enrollment into EPIC study) to CRC diagnosis was 4.8 years (interquartile range [IQR], 2.6–7.0). The median follow‐up time for our analysis, defined as the time from CRC diagnosis to the date of death or center‐specific date of cessation of follow‐up, for CRC cases with death events was 2.1 (IQR, 0.8–4.4) years, and for those without death events, it was 11.4 (IQR, 9.4–13.5) years. The numbers of cases with CRC deaths, non‐CRC deaths, deaths with unknown cause, and censored CRC cases were 515 (37.6%), 148 (10.8%), 7 (0.5%), and 701 (51.1%), respectively. Among the censored CRC cases, 692 cases were alive at the end of follow‐up, and 9 cases (7 men, 2 women) were lost to follow‐up.

FABP‐4 concentrations were on average higher in women than in men (mean ± SD in men, 13.5 ± 8.3 ng/mL, in women, 21.3 ± 9.9 ng/mL). One participant who died of CRC 5 months after diagnosis had an extreme FABP‐4 value of 154.4 ng/mL, which was more than twice as high as the second‐highest value (67.3 ng/mL). This participant had a BMI of 24.6 and WC of 99—values within the 1st to 3rd quartiles of the distribution of the index variables—but exhibited extreme levels of insulin‐like growth factor‐binding protein (IGFBP)‐1, IGFBP‐2, intact IGFBP‐3, lipocalin‐2; high levels (>97th percentile) of high‐density lipoprotein cholesterol (HDLC), high‐sensitivity C‐reactive protein (hsCRP), and Ferric reducing the ability of plasma; and very low levels (<3rd percentile) of 25‐hydroxyvitamin D and HbA1c. Tests of linearity by comparing models with and without the addition of nonlinear terms (other covariates as in Model 3) did not show any deviations from linearity, despite the presence of the extreme value. Therefore, this participant was retained in the main analyses but excluded in a sensitivity analysis. Study characteristics across quartiles of FABP‐4 are described in Table 1. The mean age of participants at diagnosis of CRC was 63 ± 7.4 years, with ~50% of CRC cases being stage I/II and 50% being stage III/IV. CRC cases in the higher versus lower quartiles of FABP‐4 were characterized by older age, higher BMI and WC measures, lower consumption of vegetables, alcohol, energy, and fiber, and higher consumption of processed meats. These CRC cases were also less likely to be smokers and more likely to have diabetes. CRC cases in the highest quartile of FABP‐4 also had higher levels of leptin, triglycerides, HbA1c, CRP, and C‐peptide; while they had lower levels of adiponectin, soluble leptin receptor, and HDLC compared with the lowest quartile. Correlations of FABP‐4 were 0.47 (95% CI: 0.42–0.51) with BMI; 0.46 (95% CI: 0.42–0.50) with WC; and 0.46 (95% CI: 0.40–0.51) with leptin; and 0.34 (95% CI: 0.28–0.39) with hsCRP (Table S2).

**TABLE 1:** Summary of baseline, prognostic and outcomes characteristics of sampled CRC cases (*N* = 1371) categorized by sex‐specific quartiles of FABP‐4 in the European Prospective Investigation into Cancer and Nutrition (EPIC) study.

	Quartile of pre‐diagnostic circulating FABP‐4 concentrations				
	1 (*N* = 323)	2 (*N* = 315)	3 (*N* = 353)	4 (*N* = 380)	Total (*N* = 1371)
FABP‐4 (ng/mL), Male	≤9.0		12.11–16.0	>16.0	
FABP‐4 (ng/mL), female	≤14.0	14.01–18.3	18.31–24.5	>24.5	
End‐point values (at the end of follow‐up)					
CRC outcome, %					
Censored	61.3	55.9	49.9	39.7	51.1
CRC death	31.9	34.9	35.7	46.3	37.6
Non‐CRC death	6.5	8.9	14.2	12.9	10.8
Death of unknown cause	0.3	0.3	0.3	1.1	0.5
Prognostic variables (at diagnosis)					
Age at CRC diagnosis, mean (SD)	60.5 (7.3)	62.6 (7.3)	63.7 (7.3)	64.8 (7.0)	63.0 (7.4)
Site of the tumor, %					
Colon	61.3	60.6	67.1	65.5	63.8
Rectum	38.7	39.4	32.9	34.5	36.2
Stage of the tumor, %					
I/II	52.1	49.6	51.8	53.1	51.8
III/IV	47.9	50.4	48.2	46.9	48.2
Baseline variables (at recruitment)					
Sex, female, %	48.6	52.1	50.4	54.5	51.5
Body mass index, mean (SD)	24.5 (3.1)	25.6 (3.4)	27.0 (3.6)	29.4 (4.4)	26.8 (4.1)
Waist circumference, mean (SD)	84.6 (11.3)	88.0 (12.0)	91.6 (11.5)	97.2 (12.7)	90.5 (12.8)
Current smoker, %	32.3	26.5	24.8	21.4	26.0
Moderately active and Active levels of the combined total physical activity index (sex‐specific categ.), %	53.2	52.3	58.4	51.3	53.8
Self‐reported diabetes or Hba1c ≥ 6.5%, %	5.1	6.0	7.2	15.3	8.7
Longer education (incl. University degree), %	20.0	19.4	18.3	14.6	17.9
Had polyps of the large bowel, %	4.2	1.9	4.7	5.9	4.2
Vegetables (g/d), median (IQR)	166.3 (109.3, 237.7)	157.8 (106.0, 244.6)	153.2 (99.6, 230.9)	144.8 (91.9, 214.8)	154.8 (101.7, 231.2)
Fruit (g/d), median (IQR)	200.5 (102.7, 291.1)	186.5 (112.6, 296.9)	173.8 (94.5, 290.5)	176.9 (81.9, 285.4)	184.8 (97.2, 289.2)
Dairy products (g/d), median (IQR)	274.0 (138.7, 461.7)	268.6 (158.3, 424.1)	284.3 (147.0, 494.0)	296.4 (153.1, 433.6)	281.5 (150.2, 455.3)
Red meat (g/d), median (IQR)	45.9 (26.2, 75.5)	48.7 (26.4, 72.9)	49.4 (22.2, 76.9)	46.1 (24.2, 76.4)	48.0 (24.6, 76.4)
Processed meat (g/d), median (IQR)	23.9 (12.6, 43.8)	25.5 (12.5, 43.7)	25.9 (14.2, 44.4)	26.3 (15.8, 46.7)	25.5 (13.7, 44.5)
Fish and shellfish (g/d), median (IQR)	28.8 (15.9, 53.1)	26.2 (14.6, 42.9)	26.4 (14.9, 47.5)	28.5 (14.9, 51.7)	27.8 (15.1, 48.8)
Alcohol (g/d), median (IQR)	19.9 (26.5)	17.3 (19.2)	16.5 (22.4)	15.8 (21.7)	17.3 (22.6)
Energy (kcal/d), mean (SD)	2224.8 (671.9)	2161.8 (673.9)	2122.4 (611.2)	2044.2 (655.1)	2134.1 (655.2)
Total dietary fiber (g/d), mean (SD)	23.9 (7.74)	23.4 (8.32)	22.7 (7.11)	21.7 (7.58)	22.9 (7.71)
Total adiponectin (μg/mL), mean (SD)	7.5 (3.5)	7.5 (3.9)	7.0 (3.6)	7.0 (3.2)	7.2 (3.6)
HMW adiponectin (μg/mL), mean (SD)	4.1 (2.6)	4.2 (2.9)	3.7 (2.6)	3.7 (2.4)	3.9 (2.6)
Leptin (ng/mL), median (IQR)	5.3 (2.7, 9.7)	7.8 (4.1, 14.1)	9.4 (5.3, 16.4)	15.4 (8.0, 28.3)	8.9 (4.6, 17.5)
Soluble leptin receptor (ng/mL), median (IQR)	21.5 (18.2, 26.6)	20.8 (17.4, 25.1)	20.5 (16.4, 24.0)	18.1 (14.5, 22.8)	20.3 (16.2, 24.5)
Total cholesterol (mmol/L), mean (SD)	6.2 (1.1)	6.4 (1.2)	6.5 (1.2)	6.6 (1.3)	6.4 (1.2)
High‐density lipoprotein cholesterol (mmol/L), mean (SD)	1.5 (0.4)	1.5 (0.4)	1.4 (0.4)	1.4 (0.4)	1.4 (0.4)
Triglycerides (mmol/L), mean (SD)	1.4 (0.8)	1.7 (1.2)	1.8 (1.1)	2.2 (1.4)	1.8 (1.2)
HbA1c (NGSP standardization) (%), mean (SD)	5.7 (0.7)	5.7 (0.5)	5.9 (0.7)	6.2 (1.2)	5.9 (0.8)
C‐reactive protein (μg/mL), median (IQR)	1.6 (0.5, 3.1)	2.2 (0.8, 3.8)	2.8 (1.1, 4.7)	4.3 (2.0, 7.2)	2.6 (1.0, 4.9)
C‐peptide (ng/mL), mean (SD)	4.0 (2.3)	4.6 (2.9)	4.7 (2.6)	5.8 (2.9)	4.8 (2.7)
Reactive oxygen metabolites (Carratelli units), mean (SD)	383.8 (70.2)	391.8 (71.2)	396.4 (79.1)	417.1 (73.3)	398.1 (74.7)

*Note*: IQR is the interquartile range, ranged between the 75th and 25th percentiles of the data. Missing data of stage, WC and diabetes were in 185 (13.5%), 75 (5.5%), and 68 (5%) participants, respectively. Wherever missing data present, the estimates were derived from the non‐missing data only.

### 3.1 Association of FABP‐4 levels with mortality after CRC

Unadjusted cumulative incidence functions (CIFs) describing the different cumulative probabilities of CRC‐specific mortality over time among 4 quartiles of FABP‐4 levels and between high and low FABP‐4 levels using the 75th percentile of FABP‐4 as a threshold are shown in Figure S4, with a statistically significant difference in the CIF of the 4th quartile compared with the CIF of the 1st, 2nd, and 3rd quartiles combined (*p*‐values for the Gray's test for homogeneity of the CIFs ≤.0001).

After multivariable adjustment, including adiposity measures (BMI and *ɛ*(WC|BMI + height)), we observed a statistically significant association of baseline FABP‐4 concentrations with the risk of CRC‐specific and all‐cause mortality (Table 2). Individuals in the highest compared to the lowest FABP‐4 quartile had a 1.49‐fold higher risk of CRC‐specific death (95% CI: 1.11–2.00; *p*‐trend = .01) and a similar higher risk of all‐cause death. On a continuous scale, 1 SD (8.9 ng/mL^14^) higher FABP‐4 concentrations were related to a 1.11‐fold higher risk of all‐cause death (95% CI: 1.02–1.20; *p* = .01). In analyses that did not adjust for adiposity measures, higher FABP‐4 concentrations were significantly associated with an increased risk of non‐CRC mortality, but this association was no longer significant after such adjustment.

**TABLE 2:** Hazard ratios (HRs) and 95% confidence intervals (CIs) for CRC‐specific and all‐cause mortality according to sex‐specific quartiles and per SD FABP‐4 in the European Prospective Investigation into Cancer and Nutrition (EPIC) study (*N* = 1371).

	1st quartile	2nd quartile	3rd quartile	4th quartile	*p*‐trend^a^	Per SD^b^	*p*‐ per SD
FABP‐4 (ng/mL)							
Male	≤9.0	9.01–12.1	12.11–16.0	>16.0			
Female	≤14.0	14.01–18.3	18.31–24.5	>24.5			
*N*	323	315	353	380		1371	
Censored^c^	198	176	176	151		701	
Primary outcome: CRC‐specific mortality							
Deaths from the main event	103	110	126	176		515	
Deaths from competing events	22	29	51	53		155	
Model 1, HR (95% CI)	ref	1.17 (0.89–1.53)	1.15 (0.89–1.50)	1.61 (1.25–2.06)	<.001	1.14 (1.06–1.23)	<.001
Model 2, HR (95% CI)	ref	1.16 (0.88–1.52)	1.15 (0.88–1.51)	1.61 (1.24–2.08)	<.001	1.13 (1.05–1.22)	.002
Model 3, HR (95% CI)	ref	1.14 (0.86–1.50)	1.11 (0.84–1.47)	1.49 (1.11–2.00)	.01	1.09 (0.99–1.20)	.07
Secondary outcome: Non‐CRC‐specific mortality							
Deaths from the main event	21	28	50	49		148	
Deaths from competing events	104	111	127	180		522	
Model 1, HR (95% CI)	ref	1.53 (0.87–2.69)	2.30 (1.37–3.85)	2.46 (1.47–4.14)	<.001	1.25 (1.12–1.39)	<.001
Model 2, HR (95% CI)	ref	1.45 (0.81–2.61)	2.01 (1.18–3.45)	2.03 (1.18–3.51)	.006	1.25 (1.07–1.44)	.004
Model 3, HR (95% CI)	ref	1.30 (0.72–2.35)	1.73 (1.00–3.00)	1.51 (0.82–2.76)	.14	1.16 (0.97–1.39)	.11
Secondary outcome: All‐cause mortality							
Total deaths	125	139	177	229		670	
Model 1, HR (95% CI)	ref	1.22 (0.96–1.55)	1.30 (1.04–1.64)	1.72 (1.38–2.14)	<.001	1.17 (1.10–1.24)	<.001
Model 2, HR (95% CI)	ref	1.20 (0.94–1.54)	1.30 (1.02–1.64)	1.69 (1.34–2.12)	<.001	1.16 (1.08–1.23)	<.001
Model 3, HR (95% CI)	ref	1.16 (0.90–1.48)	1.22 (0.95–1.55)	1.49 (1.15–1.93)	.003	1.11 (1.02–1.20)	.01

*Note*: HRs for CRC‐specific and non‐CRC‐specific mortality were estimated from cause‐specific hazards models accounting for competing risks. HRs for all‐cause mortality were estimated from conventional Cox proportional hazards models. The time function in all models was the time from CRC diagnosis to the event of death or the last follow‐up. Model 1: Stratified by country and adjusted for sex (crude model). Model 2: Model 1 and adjusted for age at diagnosis (continuous), smoking status (never, past, current smoker), education (none, primary school, technical/professional school, secondary school, or longer education), sex‐specific categories of physical activity (inactive, moderately inactive, moderately active, active), polyps of the large bowel (yes, no), alcohol consumption per day (continuous), and intake per day of red meat, processed meat, fish and shellfish, calcium, dietary fiber, dairy products, vegetables and fruits (all variables were continuous). Model 3: Model 2 and additionally adjusted for adiposity measures including body mass index (BMI) (continuous), and residuals of waist circumference (WC) when regressed on BMI and height (*ɛ*[WC|BMI and height]). Of note, data were missing in WC (75 participants (5.5%); smoking status (11 participants (0.8%)), physical activity index (87 participants (6.3%)), education (44 participants (3.2%)), polyps of the large bowel (252 participants (18.4%)), and all dietary variables (4 participants (0.3%))); and were replaced by multiple imputations.

^a^ *p*‐trend: *p* values were from testing the hypothesis of equal effects across quartiles of FABP‐4 while accounting for covariates in the model.

^b^ Estimated per one SD increment of FABP‐4 concentrations.

^c^ Censored: Cases that were either lost to follow‐up or remained alive at the end of the follow‐up in this study.

**FIGURE 2:**
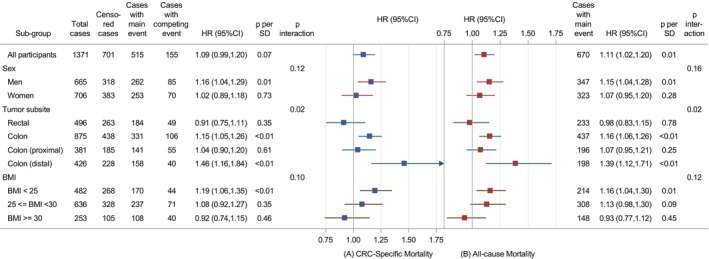
Hazard ratios (HRs) and 95% confidence intervals (CIs) for CRC‐specific and all‐cause mortality per 1 SD increment of FABP‐4 levels in the European Prospective Investigation into Cancer and Nutrition (EPIC) study (*N* = 1371), in subgroup analyses. HRs for the primary outcome (A), CRC‐specific mortality, were estimated from cause‐specific Cox proportional hazards models accounting for competing risks. HRs for the secondary outcome (B), all‐cause mortality, were estimated from conventional Cox proportional hazards models. The time function in all models was the time from CRC diagnosis to the event of death or the last follow‐up. All models were stratified by country and adjusted for sex, age at diagnosis (continuous), smoking status (never, past, current smoker), education (none, primary school, technical/professional school, secondary school, or longer education), sex‐specific categories of physical activity (inactive, moderately inactive, moderately active, active), polyps of the large bowel (yes, no), alcohol consumption per day (continuous), intake per day of red meat, processed meat, fish and shellfish, calcium, dietary fiber, dairy products, vegetables and fruits (all variables were continuous), and adiposity measures including body mass index (BMI) (continuous) and residuals of waist circumference (WC) when regressed on BMI and height (*ɛ*[WC|BMI and height]). Of note, data on stage, WC, and diabetes were missing in 185 (13.5%), 75 (5.5%), and 68 (5%) individuals, respectively. The missing data were replaced by multiple imputations. The *p*‐interaction was estimated as the *p* value of the interaction term between FABP‐4 and each of the potential effect modifiers.

The interaction terms of FABP‐4 with tumor stage, with time to CRC diagnosis, and with diabetic status all had *p*‐values >0.4 (data not shown); no further subgroup analyses were conducted for these variables. Tests for interaction terms for FABP‐4 with tumor subsite were significant for CRC‐specific and all‐cause mortality (*p* = .02 for both outcomes) (Figure 2): there were positive associations between FABP‐4 concentrations and CRC‐specific mortality (HR per SD = 1.15; 95% CI: 1.05–1.26) as well as all‐cause mortality (HR per SD = 1.16; 95% CI: 1.06–1.26) in individuals with colon cancer but not in those with rectal cancer. Higher FABP‐4 levels were associated with an increased risk of CRC‐specific mortality (HR per SD = 1.46; 95% CI: 1.16–1.84) and all‐cause mortality (HR = 1.39; 95% CI: 1.12–1.71) in distal colon cancer but not proximal colon cancer (Figure 2). No statistically significant interactions were observed between FABP‐4 and sex (*p* = .12 for CRC‐specific), or FABP‐4 and BMI (*p* = .10 for CRC‐specific; Figure 2). In sex‐stratified analyses, higher FABP‐4 levels were associated with increased CRC‐specific (HR per SD = 1.16; 95% CI: 1.04–1.29) and all‐cause mortality (HR = 1.15; 95% CI: 1.04–1.28) among men, but not among women. BMI‐stratified analyses showed associations between FABP‐4 and CRC‐specific mortality (HR per SD = 1.19; 95% CI: 1.06–1.35) as well as all‐cause mortality (HR per SD = 1.16; 95% CI: 1.04–1.30) in individuals with normal BMI (<25 kg/m^2^), but not in those with overweight (25 to <30 kg/m^2^) or obesity (≥30 kg/m^2^) (Figure 2).

In a sensitivity complete case analysis including 1046 participants (76% of the total study sample), FABP‐4 was significantly associated with both CRC‐specific mortality (HR _per SD_ = 1.10; 95% CI: 1.00–1.22; *p* = .05) and all‐cause mortality (HR _per SD_ = 1.11; 95% CI: 1.02–1.21; *p* = .02) (Table S4). We performed sensitivity analyses that excluded the one participant with the extreme FABP‐4 value in both the multiple imputation and the complete case datasets. This exclusion did not materially alter the overall results in either the multiple imputation or complete case datasets. However, in the multiple imputation data, the previously significant associations with CRC‐specific mortality in both men and in the BMI <25 group became non‐significant (Table S4). In the complete case data, the association in men was attenuated to non‐significance, but the association in the BMI <25 group remained significant (Table S4). Results from the Fine–Gray subdistribution hazard models were consistent with those from the cause‐specific Cox models (Tables S3 and S4). Scatter plots of FABP‐4 concentrations against both the time to CRC diagnosis (Figure S5) and against time from recruitment to time of laboratory analysis (Figure S6) showed zero‐slope lines.

### 3.2 Mediation effect of FABP‐4 on the association between obesity and survival after CRC

Using the counterfactual approach, the TEs of BMI on CRC‐specific, non‐CRC‐specific, and all‐cause mortality were HR_per 5 units of BMI_ = 1.18 (95% CI: 1.05–1.32); 1.32 (95% CI: 1.07–1.61); and 1.21 (95% CI: 1.10–1.34), respectively. The DEs on CRC‐specific, non‐CRC‐specific, and all‐cause mortality per 5 units higher BMI while holding the levels of FABP‐4 at a fixed level were HR = 1.11 (95% CI: 0.98–1.27); 1.19 (95% CI: 0.95–1.49); 1.13 (95% CI: 1.02–1.26), respectively. The MPs by FABP‐4 estimated using the difference method were 33.7%, 36.1%, and 34.5% of the associations between BMI with CRC‐specific mortality, non‐CRC‐specific mortality, and all‐cause mortality, respectively (Table 3). Circulating FABP‐4 concentrations alone explained 18.5% of the variation in BMI (data not shown). Using the standard product‐of‐coefficients approach, we estimated similar TEs and DEs as in the counterfactual approach, with the statistically significant IEs of BMI on all‐cause mortality (Table S5). When we dichotomized BMI into obesity (BMI ≥30 kg/m^2^) versus non‐obesity (BMI <30 kg/m^2^), the MP by FABP‐4 was higher for CRC‐specific mortality (38.5%, *p* = .005) than for non‐CRC‐specific mortality (21.9%, *p* = .03) (Table S6).

**TABLE 3:** The decompositions of the effect and mediation proportions mediated by FABP‐4 in the relationship of BMI (per 5 kg/m^2^) and mortality using the causal approach.

	The total effect (TE) and 95% CI	The direct effect (DE) and 95% CI	The mediation proportion and 95% CI of the association mediated by FABP‐4 (MP) (MP = 1 − log(HR^DE^)/log(HR^TE^))
BMI per 5 unit and the outcome			
Outcome: CRC specific mortality	1.18 (1.05–1.32)	1.11 (0.98–1.27)	33.7% (7.1%–77.1%), *p* = .03
Outcome: non‐CRC‐specific mortality	1.32 (1.07–1.61)	1.19 (0.95–1.49)	36.1% (8.2%–78.2%), *p* = .02
Outcome: All‐cause mortality	1.21 (1.10–1.34)	1.13 (1.02–1.26)	34.5% (12.6%–65.9%), *p* = .002

*Note*: The mediation analysis was performed with the causal approach and assuming no interaction between FABP‐4 and BMI using SAS Macro %mediate which was designed for time‐to‐event data with a stratified sample design. Mediation analyses were performed using the first imputed dataset. Other imputed datasets were similar and results were therefore omitted. In this analysis, we estimated the effect per 5 kg/m^2^ of BMI. The Cox proportional hazard model used in the mediation analysis has the time function in all models and was the time from CRC diagnosis to the event of death or the last follow‐up. All models were stratified by country, and adjusted for sex, age at diagnosis (continuous), smoking status (never, past, current smoker), education (none, primary school, technical/professional school, secondary school, or longer education), sex‐specific categories of physical activity (inactive, moderately inactive, moderately active, active), polyps of the large bowel (yes, no), alcohol consumption per day (continuous), intake per day of red meat, processed meat, fish and shellfish, calcium, dietary fiber, dairy products, vegetables and fruits (all variables were continuous).

## 4 DISCUSSION

In this study, we found a statistically significant positive association between pre‐diagnostic FABP‐4 concentrations and CRC‐specific and all‐cause mortality in persons with CRC, even after adjustment for BMI and WC. In subgroup analyses, the associations were apparent in persons with distal colon cancer but not in proximal colon cancer or rectal cancer. Finally, we found that FABP‐4 may mediate 33.7%, 36.1%, and 34.5% of the relationship between BMI and CRC‐specific mortality, non‐CRC‐specific mortality, and all‐cause mortality, respectively. These observations support the hypothesis that higher pre‐diagnostic FABP‐4 concentrations are related to higher mortality in persons with CRC and that FABP‐4 may be among the mediators for the relationship of obesity with mortality in these individuals.

The potential roles of FABP‐4 and the mechanisms by which FABP‐4 may be involved in cancer progression were studied in several experiments.^16^, ^17^ An in vitro study showed that incubating HCT‐8 and HCT‐116 colon cancer cells with adipose tissue extract increased the invasion and migration of colon cancer cells, which was then suppressed by the FABP‐4 inhibitor BMS309403.^17^ Further, overexpressing FABP‐4 in HCT‐8 cells using a FABP‐4 recombinant adenovirus enhanced cell migration in the wound healing assay, compared to the control.^17^ Mice injected with FABP‐4‐overexpressed HCT‐116 cells had a higher rate of lung metastases compared to the control group injected with normal HCT‐116 cells. Thus, it is suggested that FABP‐4 enhances the invasiveness, migration, and metastasis of colon cancer cells.^17^, ^34^ Interestingly, similar mechanisms may also play a role early in cancer development, as normal endothelial cells with FABP‐4 deficiency show significantly reduced migration and invasion.^16^ This suggests that FABP‐4 could contribute to tumor progression from the early stages through to metastasis. The mechanism underlying these observations includes promoting the transport and accumulation of lipids, the enhancement of energy and lipid metabolism, activating the AKT pathway, and epithelial‐mesenchymal transition.^17^ Furthermore, FABP‐4 may also promote angiogenesis through the downregulation of the P38 MAPK and eNOS pathways, and by altering SCF/c‐kit signaling.^16^ FABP‐4 may also be involved in the progression of cancer indirectly by promoting insulin resistance and inflammation,^11^ two potential pathways in which obesity is implicated in CRC progression.^35^

One may question whether collider stratification biases may have affected our results. Including only individuals diagnosed with CRC, a form of conditioning on CRC diagnosis, may act as a collider between pre‐diagnostic FABP‐4 and other CRC risk factors.^28^, ^36^ Thus, a non‐causal correlation would have been created between FABP‐4 and factors that affect the collider (CRC incidence), such as smoking, sex, education, place of residence, time from obesity onset to participants' enrollment, the intentionality of weight reduction, and genetic influences^26^ (Figure 1). In this study, we therefore adjusted for most of the potential risk factors of CRC to block any potential backdoor paths and minimize potential non‐causal correlations that could have been introduced by conditioning on CRC. Estimates from the model adjusted for all of these factors (Model 2) were not different from the crude model (Model 1). Therefore, based on our data, we speculate that any potential collider stratification biases, if introduced, were minor.

We observed increased mortality estimates for higher pre‐diagnostic circulating FABP‐4 in participants with distal colon cancer but not with proximal colon or rectal cancer, which may be due to obesity‐related inflammation interacting with the unique molecular traits of distal tumors, potentially worsening tumor aggressiveness.^5^ Furthermore, we observed a statistically significant association in men but not in women, in participants with normal‐weight (18 ≤ BMI < 25 kg/m^2^) but not with overweight or obese; however, these associations were attenuated and no longer statistically significant after excluding one participant with an extreme level of FABP‐4. The one participant with an extremely high level of FABP‐4 also showed consistently extreme levels—both high and low—of several other biomarkers across measurements taken at different time points from separate aliquots of the same biobanked sample. This pattern suggests a rare and biologically unusual profile that could substantially influence subgroup results. Including this participant captures the full variability within the population, while excluding this participant provides estimates that may better reflect typical associations in individuals with normal BMI. It is important to note that these subgroup analyses present challenges, including issues of multiple testing, low sample size, limited statistical power, and the loss of information when categorizing continuous variables, all of which can affect the robustness of results.^37^, ^38^ For example, effect estimates in these subgroups remained elevated after excluding the extreme value, with wider confidence intervals, suggesting small sample sizes and limited powers in these subgroups. The association observed in men but not in women before excluding the extreme value was similar to what was observed for risk of cardiovascular diseases (CVDs) mortality in the Cardiovascular Health Study.^39^ That study showed that higher levels of FABP‐4 were associated with higher risk of CVD mortality in men (HR per doubling FABP‐4 = 1.33; 95% CI: 1.09–1.63) but not in women (HR per doubling FABP‐4 = 1.14; 95% CI: 0.99–1.32) among participants with incident CVDs.^39^ Therefore, these subgroup findings should be interpreted cautiously and considered exploratory. Larger studies are needed to clarify whether FABP‐4—mortality association varies by sex or BMI and to confirm these observations.

As one of the most abundant proteins ever found in mature adipocytes and adipose tissue,^11^ it is not unexpected that previous EPIC analyses reported a moderate correlation between FABP‐4 and BMI, and WC in controls.^12^, ^14^ In the present study, we estimate a similar correlation for BMI, WC, and leptin (an adipokine) and baseline FABP‐4 in individuals with CRC (after a mean follow‐up of 4.98 ± 2.94 years). These findings suggest a potential role of FABP‐4 concentrations in the association of obesity/higher BMI and higher mortality in individuals with CRC. Indeed, our findings suggest that a substantial proportion of the association of BMI with mortality in individuals with CRC may be mediated by circulating FABP‐4 concentrations. In this study, we used both counterfactual and standard approaches to decompose the TEs to DE/IEs of BMI on mortality. Although the two approaches rely on different sets of assumptions and analytic means, we observe similar TEs and DEs. Thus, findings in this study suggest that FABP‐4, as an obesity‐related biomarker, may be a mediator in the BMI‐mortality relationship in persons with CRC. Experimental studies have shown that the FABP‐4 inhibitor BMS309403 could suppress the invasion and migration of colon cancer cells.^17^ Omega‐3 fatty acids may reduce circulating FABP‐4 levels by suppressing its expression in adipocytes, as has been shown in a study of dyslipidemia patients.^40^ Therefore, treatments targeting FABP‐4 may be a promising approach for managing CRC progression.^41^ Of note, there are more potential mediators for the BMI–mortality relationship. A previous study using data from the EPIC cohort suggested that some metabolic biomarkers may mediate the association between adiposity and the incidence of CRC.^42^ These biomarkers—HbA1c, soluble leptin receptor, transferrin (a marker of iron metabolism), and oxidative stress biomarkers such as reactive oxygen metabolites (ROM) and ferric reducing ability of plasma (FRAP)—may also serve as potential mediators of the relationship between adiposity and mortality in individuals with CRC.^42^ Furthermore, we cannot rule out that future research may identify additional obesity‐related biomarkers that help explain the BMI–mortality relationship, but currently, there is little evidence that known obesity biomarkers are associated with mortality in CRC patients. Future studies should also include the investigation of these factors to help explain this relationship further.

The study's strengths include its prospective design, long follow‐up that facilitated mortality risk estimation, and comprehensive data on potential confounders. In addition, most of the study centers had a population‐based design, and our study includes a large sample size across several Western European countries. Due to the precious nature of biospecimens, only a subset of all CRC cases from the EPIC study was included in our analysis. We compared the characteristics of our study participants with participants in an analysis of 3859 incident CRC cases,^43^ and an analysis of 3924 incident CRC cases using data ^5^ from the EPIC cohort, which did not require biospecimen data and detected no difference in the study characteristics. Our findings may therefore be generalizable to populations with the same characteristics as the CRC cases in the EPIC cohort. Additionally, we used data from incident CRC cases, which allowed us to avoid survival bias often occurring in data with prevalent cancer cases. This bias can lead to overestimated survival rates and underestimated risks because it excludes individuals who died after the CRC incidence but before study enrollment.^44^ Further, we used pre‐diagnostic FABP‐4 levels to help avoid the influence of treatment on the estimates and to minimize reverse causation bias, as advanced cancer (the main cause of high mortality) can lead to weight loss, which may alter post‐diagnosis FABP‐4 levels.^28^ Furthermore, only pre‐diagnostic (but not post‐diagnostic) obesity has been associated with a higher risk of CRC‐specific mortality.^3^ Thus, our research question focused more on the mechanism underlying the association between pre‐diagnostic obesity and mortality, making the assessment of pre‐diagnostic FABP‐4 as an obesity biomarker particularly relevant.

Our study had some limitations. First, our study was conducted only in European populations. Therefore, its findings should not be generalized to other populations of different ethnicity (e.g., Asian, African). Second, we had only a one‐time measure of FABP‐4 levels, and FABP‐4 may change because of weight change.^28^ In a sub‐sample of EPIC‐Potsdam, good reliability of FABP‐4 measurements at four‐month intervals has been demonstrated.^45^ One may question whether FABP‐4 levels were higher in individuals with blood collected closer to the CRC diagnosis dates. However, our data indicated that FABP‐4 levels, measured once per individual, did not show significant variation between individuals, regardless of how long the measurement was taken before CRC diagnosis. We further estimated the association in the individuals whose blood samples were collected less than 2 years, 2–8 years, and 8 years or longer before CRC diagnosis; however, we observed similar estimates among these groups (Figure 2).

In conclusion, we observed that higher pre‐diagnostic FABP‐4 concentrations were associated with higher CRC‐specific and all‐cause mortality in individuals with CRC. The association between BMI and mortality may be partially explained by the pathway through FABP‐4, but there may be more mediator pathways (related to inflammation and hyperinsulinemia) that warrant further research.

## AUTHOR CONTRIBUTIONS

**Thu Thi Pham:** Conceptualization; formal analysis; validation; investigation; visualization; writing – original draft; project administration; writing – review and editing; methodology; data curation. **Katharina Nimptsch:** Conceptualization; data curation; validation; methodology; writing – review and editing. **Krasimira Aleksandrova:** Conceptualization; writing – review and editing. **Mazda Jenab:** Conceptualization; writing – review and editing. **Veronika Fedirko:** Writing – review and editing; conceptualization. **Anja Olsen:** Resources; writing – review and editing. **Anne Tjønneland:** Resources; writing – review and editing. **Claire Cadeau:** Resources; writing – review and editing. **Gianluca Severi:** Resources; writing – review and editing. **Matthias B. Schulze:** Resources; writing – review and editing. **Renée Turzanski Fortner:** Resources; writing – review and editing. **Verena Katzke:** Resources; writing – review and editing. **Claudia Agnoli:** Resources; writing – review and editing. **Carlotta Sacerdote:** Resources; writing – review and editing. **Rosario Tumino:** Resources; writing – review and editing. **Simona Signoriello:** Resources; writing – review and editing. **Camino Trobajo‐Sanmartín:** Writing – review and editing; resources. **Jesús‐Humberto Gómez:** Resources; writing – review and editing. **María‐Dolores Chirlaque:** Writing – review and editing; resources. **Maria‐Jose Sánchez:** Resources; writing – review and editing. **Marta Crous‐Bou:** Writing – review and editing; resources. **Anne May:** Resources; writing – review and editing. **Alicia Heath:** Writing – review and editing; resources. **Dagfinn Aune:** Resources; writing – review and editing. **Elisabete Weiderpass:** Resources; writing – review and editing. **Tobias Pischon:** Conceptualization; supervision; investigation; methodology; writing – review and editing.

## FUNDING INFORMATION

The coordination of EPIC is financially supported by the International Agency for Research on Cancer (IARC) and also by the Department of Epidemiology and Biostatistics, School of Public Health, Imperial College London, which has additional infrastructure support provided by the NIHR Imperial Biomedical Research Centre (BRC).

The national cohorts are supported by: Danish Cancer Society; Ligue Contre le Cancer, Institut Gustave Roussy, Mutuelle Générale de l'Education Nationale, Institut National de la Santé et de la Recherche Médicale (INSERM) (France); German Cancer Aid, German Cancer Research Center (DKFZ), German Institute of Human Nutrition Potsdam‐Rehbruecke (DIfE), Federal Ministry of Education and Research (BMBF) (Germany); Associazione Italiana per la Ricerca sul Cancro‐AIRC‐Italy, Compagnia di SanPaolo and National Research Council (Italy); Dutch Ministry of Public Health, Welfare and Sports (VWS), Netherlands Cancer Registry (NKR), LK Research Funds, Dutch Prevention Funds, Dutch ZON (Zorg Onderzoek Nederland), World Cancer Research Fund (WCRF), Statistics Netherlands (The Netherlands); Health Research Fund (FIS)—Instituto de Salud Carlos III (ISCIII), Regional Governments of Andalucía, Asturias, Basque Country, Murcia and Navarra, and the Catalan Institute of Oncology—ICO (Spain); Swedish Cancer Society, Swedish Research Council and County Councils of Skåne and Västerbotten (Sweden); Cancer Research UK (14136 to EPIC‐Norfolk; C8221/A29017 to EPIC‐Oxford), Medical Research Council (1000143 to EPIC‐Norfolk; MR/M012190/1 to EPIC‐Oxford) (United Kingdom).

## CONFLICT OF INTEREST STATEMENT

The authors declare no conflict of interest.

## ETHICS STATEMENT

Informed consent was obtained from all participants involved in the study. The EPIC study was conducted according to the guidelines of the Declaration of Helsinki and approved by the Ethical Review Board of the International Agency for Research on Cancer (IARC) and the ethical committees of the participating centers.

## Supporting information


**Data S1.** Supporting Information.

## Data Availability

The data that support the findings of this study are available from the corresponding author upon reasonable request.
