# Supplementary material for: Circulating fatty acid binding protein 4 (FABP‐4) concentrations and mortality in individuals with colorectal cancer in the European Prospective Investigation into Cancer and Nutrition study
Source: Int J Cancer. 2025 Aug 26;158(3):546–59. doi: 10.1002/ijc.70090 (PMC12670333; doi:10.1002/ijc.70090)
Supplement: Supplementary file 1 — Data S1. Supporting Information. [file IJC-158-546-s001.pdf]

Circulating fatty acid binding protein 4 (FABP-4) concentrations and mortality after CRC diagnosis in the European Prospective Investigation into Cancer and Nutrition study

Author information

Thu Thi Pham, Katharina Nimptsch, Krasimira Aleksandrova, Mazda Jenab, Veronika Fedirko, Anja Olsen, Anne Tjønneland, Claire Cadeau, Gianluca Severi, Matthias B. Schulze, Renée Turzanski Fortner, Verena Katzke, Claudia Agnoli, Carlotta Sacerdote, Rosario Tumino, Simona Signoriello, Camino Trobajo-Sanmartín, Jesús-Humberto Gómez, María-Dolores Chirlaque, Maria-Jose Sánchez, Marta Crous-Bou, Anne May, Alicia Heath, Dagfinn Aune, Elisabete Weiderpass, and Tobias Pischon

Table of contents

Supplementary data and method 2

Supplementary Figures 3

Supplementary Figure 1. Distribution of FABP-4 levels by country 3

Supplementary Figure 2. Relationship of FABP-4 levels with CRC-specific mortality, non-CRC-specific mortality, and all-cause mortality, modelled using restricted cubic splines* 4

Supplementary Figure 3. Test of the Cox proportional hazard assumption: Scaled Schoenfeld residuals over follow-up time plots 6

Supplementary Figure 4. Unadjusted cumulative incidence functions (CIFs) describing the different cumulative probabilities of CRC-specific mortality over time among 4 quartiles of FABP-4 levels and between high and low FABP-4 levels using the 75th percentile of FABP-4 as a threshold 8

Supplementary Figure 5. Scatter plot of FABP-4 levels by time from recruitment to CRC diagnosis among incident CRC cases. 9

Supplementary Figure 6. Scatter plot of FABP-4 levels by time from recruitment to time of laboratory analysis (19/03/2018). 10

Supplementary Tables 11

Supplementary Table 1. Results of the likelihood ratio test for the addition of non-linear terms of FABP4 to Model 3 with the CRC-specific mortality as the outcome 11

Supplementary Table 2. Partial Spearman's correlations* between FABP-4 and other biomarkers in incident CRC cases in the European Prospective Investigation into Cancer and Nutrition (EPIC) study 12

Supplementary Table 3. Hazard ratios (HRs) and 95% confidence intervals (CIs) for CRC-specific and non-CRC-specific mortality according to sex-specific quartiles and per SD FABP-4 in the European Prospective Investigation into Cancer and Nutrition (EPIC) study (N=1371) using the sub-distribution hazards Cox model as another approach for competing risk survival analysis (**Fine-Gray model**). 13

Supplementary Table 4. Hazard ratios (HRs) and 95% confidence intervals (CIs) for CRC-specific mortality according to sex-specific quartiles and per SD of FABP-4 in the European Prospective Investigation into Cancer and Nutrition (EPIC) study in **sensitivity sub-group analyses** ...14

Supplementary Table 5. Estimates of the direct effect (DE) and indirect effect (IE) from total effect (TE) from mediation analyses of FABP-4 as a mediator in the association between pre-diagnosis BMI and mortality using the product-of-coefficients approach (the sensitivity mediation analysis). 16

Supplementary Table 6. The decompositions of the effect and mediation proportions mediated by FABP-4 in the relationship of **obesity (yes/no)*** and mortality using the causal approach....16

Supplementary data and method

The multiple imputation procedure

As recommended by Madley-Dowd and colleagues (2019), imputed variables were

- **Covariates in the analysis model:** (age at diagnosis (continuous), smoking status (never, past, current smoker), education (none, primary school, technical/professional school, secondary school, or longer education), sex-specific categories of physical activity (inactive, moderately inactive, moderately active, active), alcohol consumption (continuous), and intake of red meat, processed meat, fish and shellfish, fruits and vegetables (all variables were continuous), body mass index (BMI) (continuous), and residuals of waist circumference (WC) when regressed on BMI and height (ε(WC| BMI and height))), follow-up time, and outcome after CRC (censored, death due to CRC, death due to other cause)
- **Auxiliary variables:** self-reported or diagnosed diabetes (yes/no), alcohol lifetime pattern (categorical: never drinkers/ former light drinkers/ former heavy drinkers/ light drinkers/ never heavy drinkers/ periodically heavy drinkers/ always heavy drinkers), cancer subsite (colon/ rectum), stage (localized/localized with invasion/ metastatic regional/ metastatic distant), grade (well differentiated/ moderately differentiated/ poorly/un-differentiated), waist circumference (continuous), total energy intake (kcal/day), total dietary fiber intake (gram/day), c-reactive protein (ng/ml), c-peptide (ng/ml), triglycerides (mmol/l), high-density lipoprotein cholesterol (mmol/l), and intake of dairy products (gram/day).

Supplementary Figures

Supplementary Figure 1. Distribution of FABP-4 levels by country

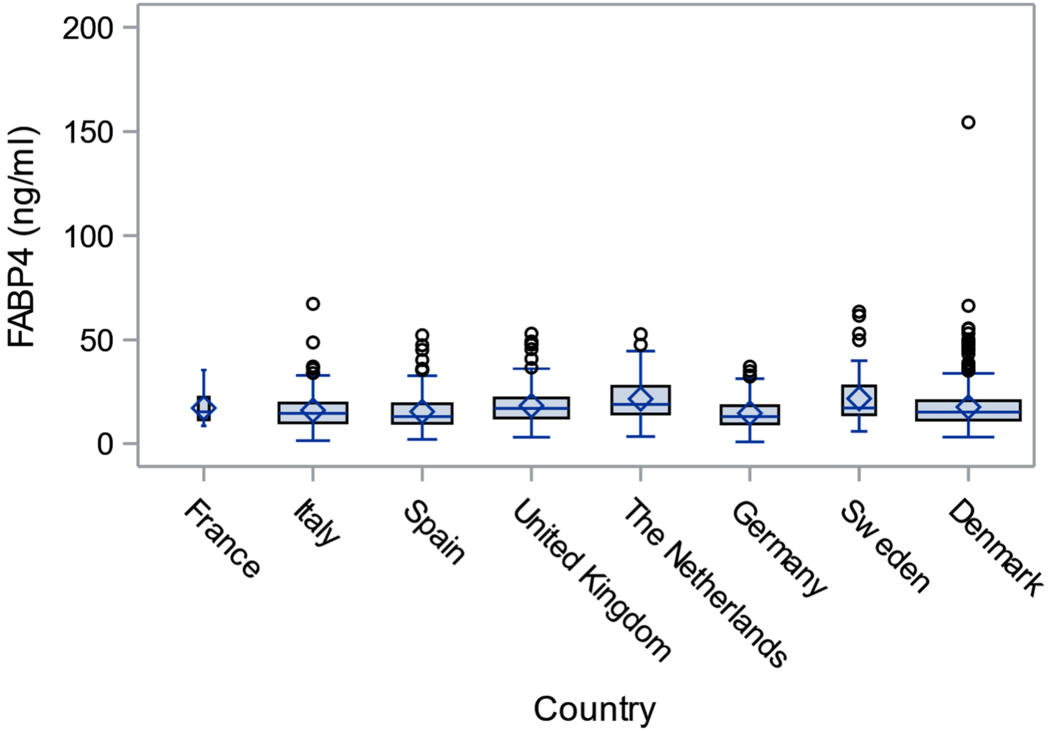

Supplementary Figure 2. Relationship of FABP-4 levels with CRC-specific mortality, non-CRC-specific mortality, and all-cause mortality, modelled using restricted cubic splines*

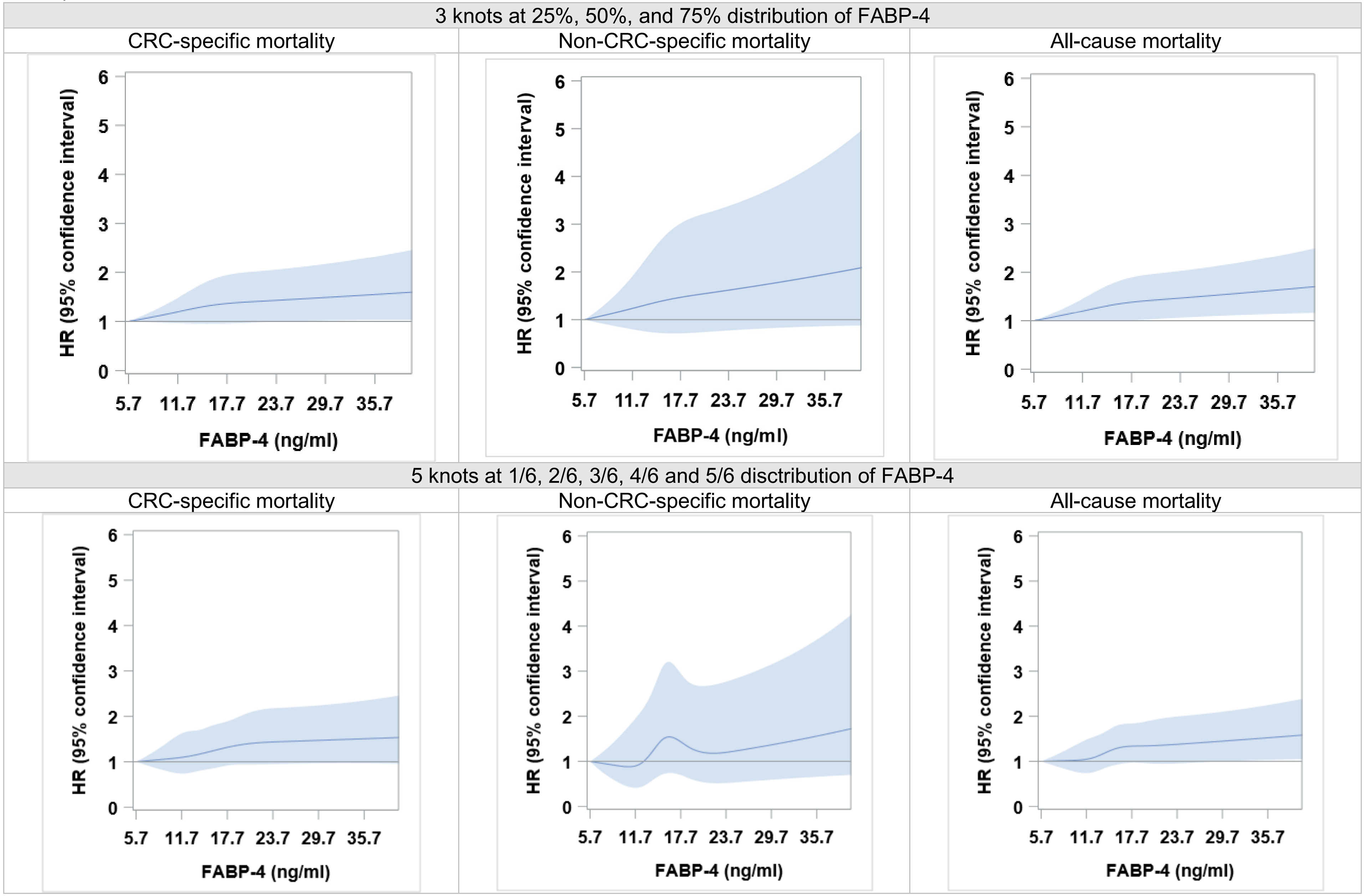

HRs for the primary outcome (CRC-specific mortality) were estimated from cause-specific Cox proportional hazards models accounting for competing risks. HRs for the secondary outcome (all-cause mortality) were estimated from conventional Cox proportional hazards models. The time function in all models was the time from CRC diagnosis to the event of death or the last follow-up. Al models were stratified by country, and adjusted for sex, age at diagnosis (continuous), smoking status (never, past, current smoker), education (none, primary school, technical/professional school, secondary school, or longer education), sex-specific categories of physical activity (inactive, moderately inactive, moderately active, active), polyps of the large bowel (yes, no), alcohol consumption per day (continuous), intake per day of red meat, processed meat, fish and shellfish, calcium, dietary fibre, dairy products, vegetables and fruits (all variables were continuous), and adiposity measures including body mass index (BMI) (continuous), and residuals of waist circumference (WC) when regressed on BMI and height (ε(WC| BMI and height). When outcomes were CRC-specific mortality and non-CRC-specific mortality, cause-specific Cox proportional hazards models were used to account for competing risks.

Supplementary Figure 3. Test of the Cox proportional hazard assumption: Scaled Schoenfeld residuals over follow-up time plots

CRC-specific mortality	All-cause mortality

Beta(t) for FABP-4 over follow-up time plots

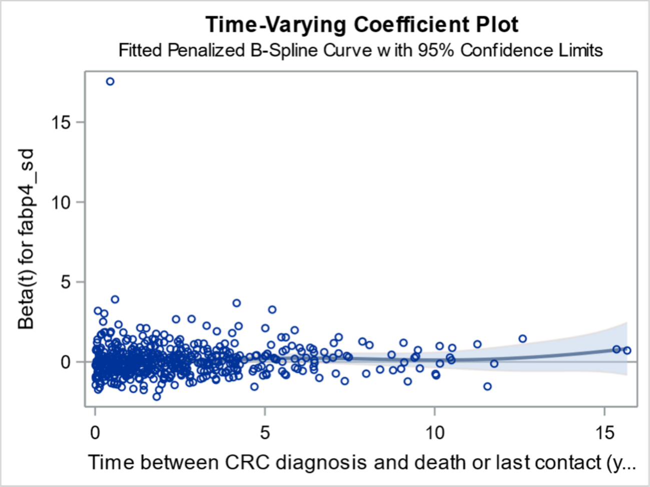

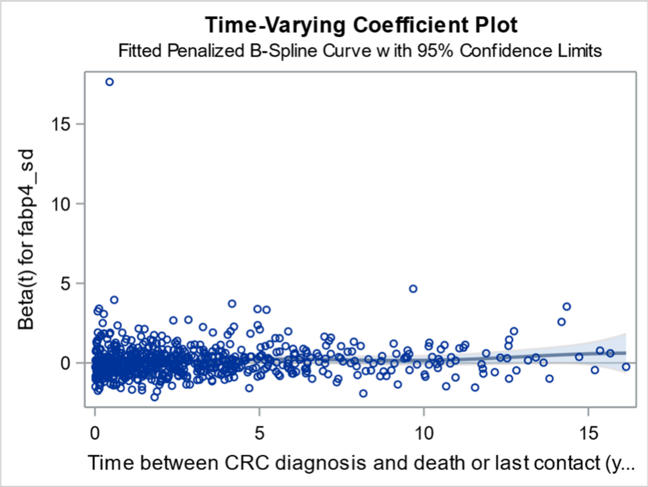

Beta(t) for BMI over follow-up time plots

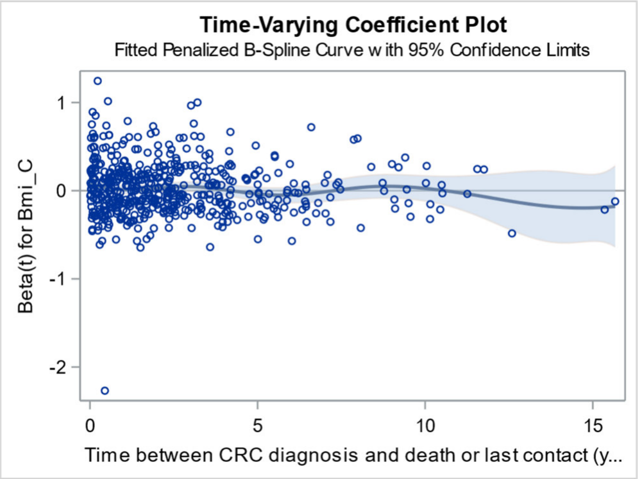

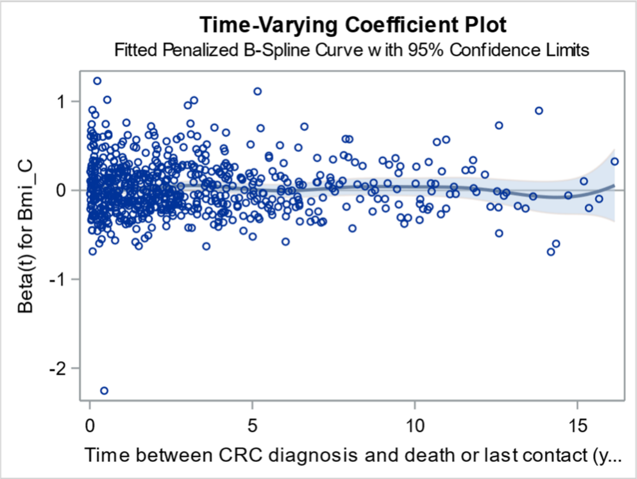

Tests were performed using the first imputed dataset; other imputed datasets were similar and thus omitted. The graphs were created by using the procedure PROC PHREG in SAS, with the zph statement. Scaled Schoenfeld residuals are independent of time, with the flatness of the beta coefficient of FABP-4 and BMI over the follow-up time suggesting proportionality of the hazards. HRs for the primary outcome (CRC-specific mortality) were estimated from cause-specific Cox

proportional hazards models accounting for competing risks. HRs for the secondary outcome (all-cause mortality) were estimated from conventional Cox proportional hazards models. The time function in all models was the time from CRC diagnosis to the event of death or the last follow-up. All models were stratified by country, and adjusted for sex, age at diagnosis (continuous), smoking status (never, past, current smoker), education (none, primary school, technical/professional school, secondary school, or longer education), sex-specific categories of physical activity (inactive, moderately inactive, moderately active, active), polyps of the large bowel (yes, no), alcohol consumption per day (continuous), intake per day of red meat, processed meat, fish and shellfish, calcium, dietary fibre, dairy products, vegetables and fruits (all variables were continuous), and adiposity measures including body mass index (BMI) (continuous), and residuals of waist circumference (WC) when regressed on BMI and height (ε(WC| BMI and height)).

Supplementary Figure 4. Unadjusted cumulative incidence functions (CIFs) describing the different cumulative probabilities of CRC-specific mortality over time among 4 quartiles of FABP-4 levels and between high and low FABP-4 levels using the 75th percentile of FABP-4 as a threshold

Cumulative probabilities of CRC-specific mortality over time among 4 quartiles of FABP-4 levels	Cumulative probabilities of CRC-specific mortality between high and low FABP-4 levels using the 75th percentile of FABP-4 as a threshold

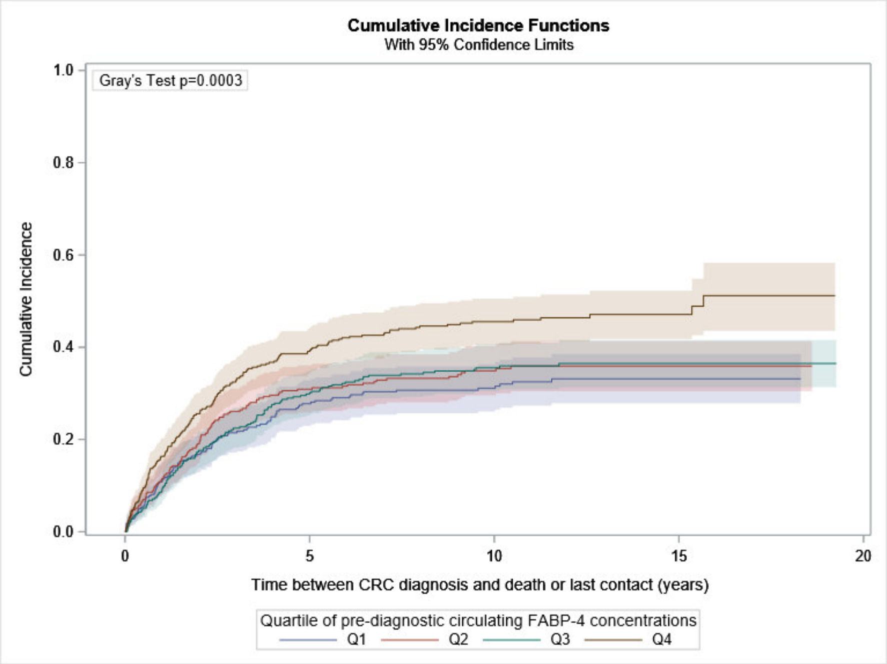

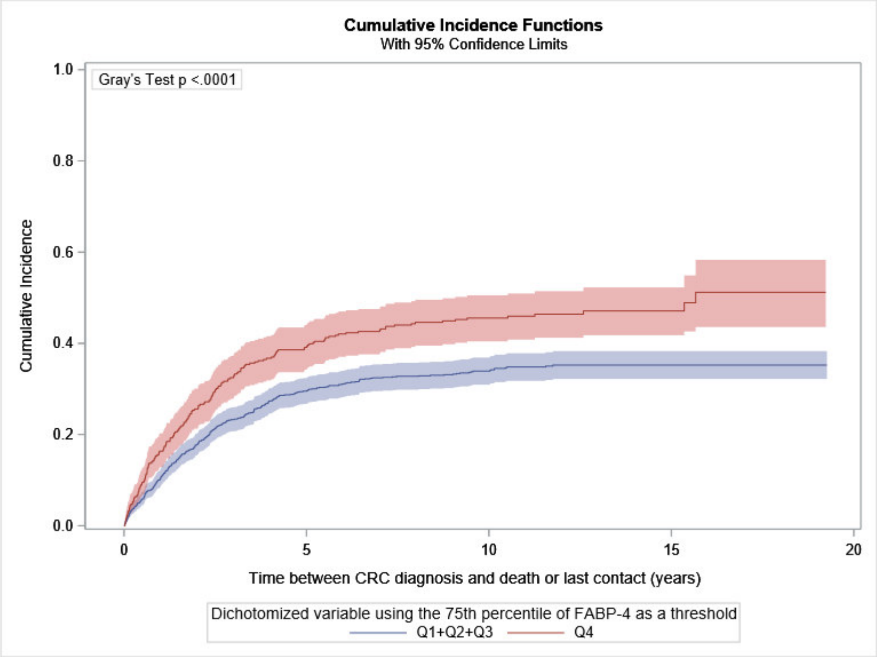

Supplementary Figure 5. Scatter plot of FABP-4 levels by time from recruitment to CRC diagnosis among incident CRC cases.

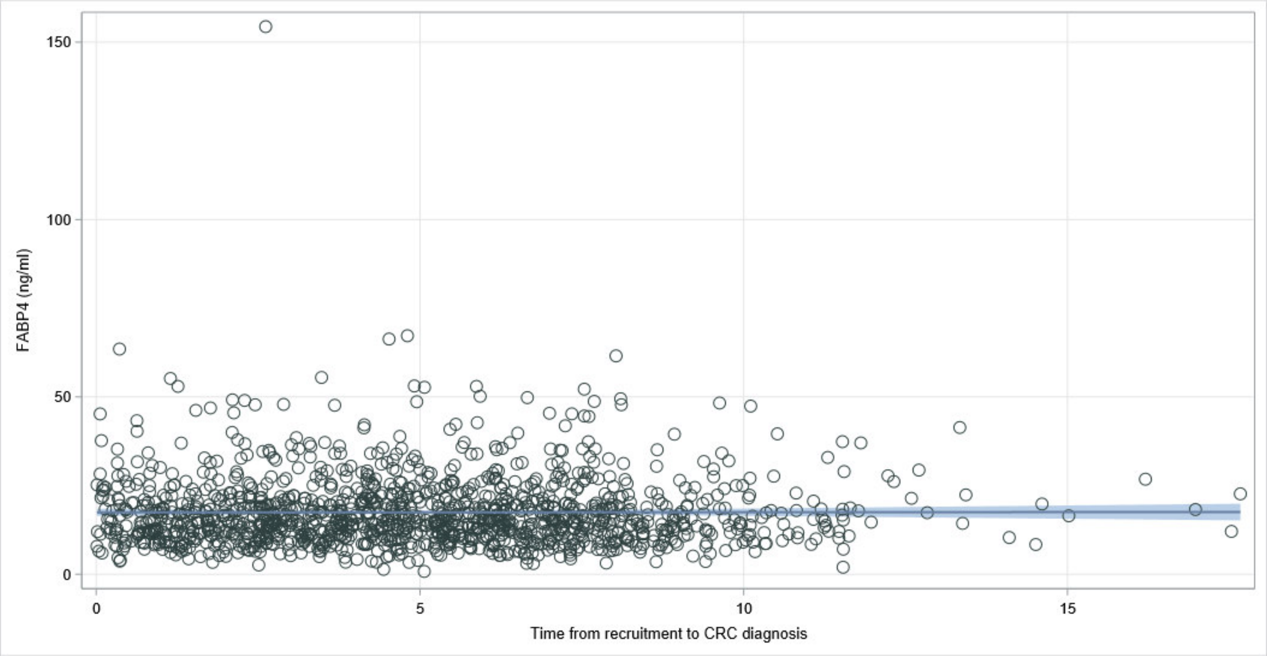

The graph illustrates the changes in FABP-4 levels when measured near the diagnosis of CRC (left) and far from the diagnosis of CRC (right). A p-value of 0.9561 was obtained for the test with the null hypothesis that the slope of the line (FABP4 ~ Time to CRC diagnosis) is equal to 0.

Supplementary Figure 6. Scatter plot of FABP-4 levels by time from recruitment to time of laboratory analysis (19/03/2018).

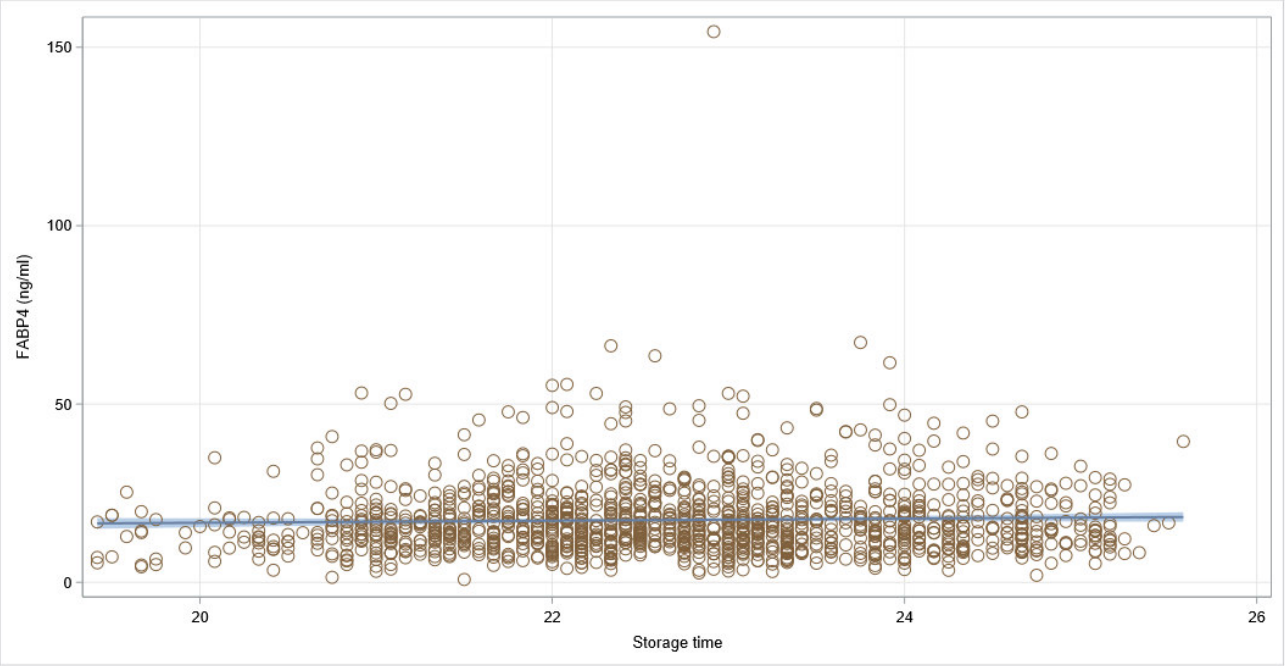

The graph illustrates the changes in FABP-4 levels against the storage time (years, defined as time from recruitment to time of laboratory analysis). A p-value of 0.1777 was obtained for the test with the null hypothesis that the slope of the line (FABP4 ~ the storage time) is equal to 0.

Supplementary Tables

Supplementary Table 1. Results of the likelihood ratio test for the addition of non-linear terms of FABP4 to Model 3 with the CRC-specific mortality as the outcome.

The non-linear term of FABP-4	Statistics comparing the reduced model and the full model *		
	Likelihood Ratio	df	p_value
cubic spline: 3 knots<br>(Places knots at the percentiles 1/4, 2/4, 3/4)	2.1795	1	0.14
cubic spline: 4 knots<br>(Places knots at the percentiles 1/5, 2/5, 3/5, 4/5)	2.9241	2	0.23
cubic spline: 5 knots<br>(Places knots at the percentiles 1/6, 2/6, 3/6, 4/6, 5/6)	2.7884	3	0.43
cubic spline: 6 knots<br>(Places knots at the percentiles 1/7, 2/7, 3/7, 4/7, 5/7, 6/7)	4.1246	4	0.39
Polynomial 2 degree	0.003	1	0.96

Tests were performed using the first imputed dataset; other imputed datasets were similar and thus omitted. * Statistics were from likelihood ratio tests comparing the likelihoods of two nested models, one with only a linear term (the reduced model) and another with both linear and non-linear terms (the full model). The insignificance of the tests suggested no evidence of a non-linear relationship. **Linearity test**: We compared model 3 to a model adding a restricted cubic spline term to model 3 ("model 3a"). Likelihood ratio (LR) tests were performed by computing the chi-squares (=abs($\mathrm{chi.square}_{\mathrm{model\ 3}}$ - $\mathrm{chi.square}_{\mathrm{model\ 3a}}$)) and the degree of freedoms (=abs($\mathrm{df}_{\mathrm{model\ 3}} - \mathrm{df}_{\mathrm{model\ 3a}}$)) [1, 2]. The p-values were then estimated under the given chi-square distribution and degree of freedom. Ref. [1] Allison, P. D. 2010. Survival Analysis Using the SAS System: A Practical Guide, Second Edition. Cary, NC: SAS Institute Inc. [2] Kleinbaum, D. G. and Klein, M. 2005. Survival Analysis: A Self-Learning Text, Second Edition. New York: Springer-Verlag.

Supplementary Table 2. Partial Spearman's correlations* between FABP-4 and other biomarkers in incident CRC cases in the European Prospective Investigation into Cancer and Nutrition (EPIC) study

Variable	Number of participants with available data	Correlation coefficients (95%CI)
Body Mass Index (computed)	1371	0.47 (0.42, 0.51)
Waist circumference (computed)	1296	0.46 (0.42, 0.50)
Adiponectin (µg/ml)	841	-0.13 (-0.19; -0.06)
HMW Adiponectin (µg/ml)	841	-0.15 (-0.22; -0.09)
Leptin (ng/ml)	841	0.46 (0.40, 0.51)
Soluble leptin receptor (ng/ml)	841	-0.21 (-0.28; -0.15)
Total cholesterol (mmol/l)	942	0.09 (0.02, 0.15)
High-density lipoprotein cholesterol (mmol/l)	936	-0.19 (-0.25; -0.13)
Triglycerides (mmol/l)	923	0.29 (0.23, 0.35)
HBA1c (NGSP standardization) (%)	836	0.20 (0.13, 0.26)
high-sensitivity C-reactive protein (µg/ml)	938	0.34 (0.28, 0.39)
C-peptide (ng/ml)	857	0.26 (0.20, 0.33)
Reactive oxygen metabolites (Carratelli units)	942	0.17 (0.11, 0.23)

All estimates were adjusted for age at diagnosis and sex.

Supplementary Table 3. Hazard ratios (HRs) and 95% confidence intervals (CIs) for CRC-specific and non-CRC-specific mortality according to sex-specific quartiles and per SD FABP-4 in the European Prospective Investigation into Cancer and Nutrition (EPIC) study (N=1371) using the sub-distribution hazards Cox model as another approach for competing risk survival analysis (**Fine-Gray model**).

		1st quartile	2nd quartile	3rd quartile	4th quartile	p-trend [a]	Per SD [b]	p-per SD
FABP-4 (ng/ml)	Male	≤9.00	9.01 to 12.38	12.39 to 16.30	≥16.30			
	Female	≤14.47	14.48 to 19.29	19.30 to 25.87	≥25.88			
N		323	315	353	380		1371	
Censored [c]		198	176	176	151		701	
Primary outcome: CRC-specific mortality								
Deaths from the main event		103	110	126	176		515	
Deaths from competing events		22	29	51	53		155	
Model 1, HR (95%CI)		ref	1.14 (0.87; 1.49)	1.09 (0.84; 1.42)	1.56 (1.22; 2.00)	<0.001	1.13 (1.06; 1.21)	<0.001
Model 2, HR (95%CI)		ref	1.13 (0.86; 1.50)	1.10 (0.84; 1.44)	1.58 (1.22; 2.06)	<0.001	1.12 (1.05; 1.20)	<0.001
Model 3, HR (95%CI)		ref	1.12 (0.84; 1.48)	1.06 (0.81; 1.40)	1.47 (1.09; 1.99)	0.02	1.09 (0.99; 1.19)	0.07
Secondary outcome: Non-CRC-specific mortality								
Deaths from the main event		21	28	50	49		148	
Deaths from competing events		104	111	127	180		522	
Model 1, HR (95%CI)		ref	1.41 (0.80; 2.47)	2.24 (1.34; 3.73)	2.05 (1.23; 3.42)	0.001	1.14 (1.03; 1.26)	0.01
Model 2, HR (95%CI)		ref	1.29 (0.73; 2.28)	2.00 (1.19; 3.39)	1.78 (1.04; 3.04)	0.01	1.18 (1.06; 1.32)	0.003
Model 3, HR (95%CI)		ref	1.18 (0.66; 2.08)	1.73 (1.01; 2.96)	1.40 (0.78; 2.52)	0.17	1.12 (0.98; 1.28)	0.11

HRs for the mortality were estimated from a sub-distribution hazards Cox model as another approach for competing risk survival analysis (Fine-Gray model). HRs for all-cause mortality) were estimated from conventional Cox proportional hazards models. The time function in all models was the time from CRC diagnosis to the event of death or the last follow-up. Covariates in each model:

Model 1: Stratified by country and adjusted for sex (crude model).

Model 2: Model 1 and adjusted for age at diagnosis (continuous), smoking status (never, past, current smoker), education (none, primary school, technical/professional school, secondary school, or longer education), sex-specific categories of physical activity (inactive, moderately inactive, moderately active, active), polyps of the large bowel (yes, no), alcohol consumption per day (continuous), and intake per day of red meat, processed meat, fish and shellfish, calcium, dietary fiber, dairy products, vegetables and fruits (all variables were continuous).

Model 3: Model 2 and additionally adjusted for adiposity measures, including body mass index (BMI) (continuous), and residuals of waist circumference (WC) when regressed on BMI and height (ε(WC| BMI and height)).

Of note, data were missing in WC (75 participants (5.5%); smoking status (11 participants (0.8%)), physical activity index (87 participants (6.3%)), education (44 participants (3.2%)), and all dietary variables (4 participants (0.3%)); and were replaced by multiple imputations.

[a]p-trend: p-values were from testing the hypothesis of equal effects across quartiles of FABP-4 while accounting for covariates in the model.
[b]Estimated by one SD FABP-4 concentration. [c]Censored: Cases that either were lost to follow-up or remained alive at the end of the follow-up in this study.

Supplementary Table 4. Hazard ratios (HRs) and 95% confidence intervals (CIs) for CRC-specific mortality according to sex-specific quartiles and per SD of FABP-4 in the European Prospective Investigation into Cancer and Nutrition (EPIC) study in **sensitivity sub-group analyses**

Sub-group	Categories	FABP-4	Model	Multiple Imputation data		Complete case analysis	
				All participants	Excluding one participant with extreme FABP-4 level (*)	All participants	Excluding one participant with extreme FABP-4 level (*)
				HR (95%CI)	HR (95%CI)	HR (95%CI)	HR (95%CI)
				(N=1371)	(N=1370)	(N=1046)	(N=1045)
CRC-specific mortality, Cause-specific model							
All participants	All participants	Q1	CS model	Ref	Ref	Ref	Ref
		Q2	CS model	1.14 (0.86, 1.50)	-	1.15 (0.85, 1.57)	-
		Q3	CS model	1.11 (0.84, 1.47)	-	1.21 (0.89, 1.66)	-
		Q4	CS model	1.49 (1.11, 2.00)	1.48 (1.10, 1.98)	1.64 (1.18, 2.28)	1.62 (1.16, 2.26)
		Per SD	CS model	1.09 (0.99, 1.20)	1.06 (0.95, 1.18)	1.10 (1.00, 1.22)	1.08 (0.95, 1.23)
Sex	Men	Per SD	CS model	1.16 (1.04, 1.29)	1.15 (0.96, 1.39)	1.16 (1.03, 1.30)	1.18 (0.94, 1.48)
	Women	Per SD	CS model	1.02 (0.89, 1.18)	-	1.04 (0.89, 1.22)	-
Tumor subsite	Colon	Per SD	CS model	1.15 (1.05, 1.26)	1.17 (1.02, 1.35)	1.16 (1.06, 1.27)	1.25 (1.05, 1.48)
	Colon (distal)	Per SD	CS model	1.46 (1.16, 1.84)	-	1.38 (1.07, 1.77)	-
	Colon (proximal)	Per SD	CS model	1.04 (0.90, 1.20)	0.92 (0.72, 1.16)	1.09 (0.96, 1.25)	1.10 (0.81, 1.50)
	Rectal	Per SD	CS model	0.91 (0.75, 1.11)	-	0.91 (0.73, 1.13)	-
BMI	BMI < 25	Per SD	CS model	1.19 (1.06, 1.35)	1.21 (0.95, 1.55)	1.19 (1.06, 1.35)	1.40 (1.03, 1.89)
	25 <= BMI <30	Per SD	CS model	1.08 (0.92, 1.27)	-	1.13 (0.93, 1.37)	-
	BMI >= 30	Per SD	CS model	0.92 (0.74, 1.15)	-	0.82 (0.63, 1.08)	-
CRC-specific mortality, Fine-Gray model							
All participants	All participants	Q1	CS model	Ref	Ref	Ref	Ref
	All participants	Q2	F&G model	1.12 (0.84, 1.48)	1.11 (0.84, 1.48)	1.13 (0.82, 1.54)	-
	All participants	Q3	F&G model	1.06 (0.81, 1.40)	1.06 (0.80, 1.40)	1.16 (0.85, 1.59)	1.16 (0.85, 1.58)
	All participants	Q4	F&G model	1.47 (1.09, 1.99)	1.46 (1.08, 1.97)	1.58 (1.12, 2.22)	1.56 (1.11, 2.20)
	All participants	Per SD	F&G model	1.09 (0.99, 1.19)	1.06 (0.95, 1.18)	1.10 (1.01, 1.20)	1.07 (0.94, 1.21)
Sex	Men	Per SD	F&G model	1.16 (1.08, 1.25)	1.16 (0.96, 1.39)	1.16 (1.08, 1.24)	1.17 (0.95, 1.45)
	Women	Per SD	F&G model	1.02 (0.89, 1.17)	-	1.03 (0.88, 1.22)	-
Tumor subsite	Colon	Per SD	F&G model	1.13 (1.06, 1.22)	1.15 (0.99, 1.33)	1.15 (1.07, 1.23)	1.20 (1.01, 1.43)
	Colon (distal)	Per SD	F&G model	1.45 (1.13, 1.85)	-	1.36 (1.05, 1.77)	-
	Colon (proximal)	Per SD	F&G model	1.03 (0.91, 1.18)	0.91 (0.71, 1.17)	1.08 (1.00, 1.18)	1.09 (0.79, 1.51)
	Rectal	Per SD	F&G model	0.92 (0.76, 1.10)	-	0.91 (0.74, 1.12)	-
BMI	BMI < 25	Per SD	F&G model	1.20 (1.11, 1.30)	1.22 (0.95, 1.56)	1.20 (1.10, 1.31)	1.39 (1.01, 1.90)
	25 <= BMI <30	Per SD	F&G model	1.07 (0.92, 1.26)	-	1.12 (0.91, 1.37)	-
	BMI >= 30	Per SD	F&G model	0.93 (0.74, 1.17)	-	0.84 (0.65, 1.09)	-
All-cause mortality							
All participants	All participants	Q1	Standard Cox	Ref	Ref	Ref	Ref
		Q2	Standard Cox	1.16 (0.90, 1.48)	-	1.18 (0.89, 1.55)	-
		Q3	Standard Cox	1.22 (0.95, 1.55)	-	1.30 (0.99, 1.71)	-
		Q4	Standard Cox	1.49 (1.15, 1.93)	1.48 (1.14, 1.92)	1.66 (1.24, 2.23)	1.65 (1.23, 2.21)
		Per SD	Standard Cox	1.11 (1.02, 1.20)	1.09 (0.99, 1.20)	1.11 (1.02, 1.21)	1.10 (0.99, 1.23)
Sex	Men	Per SD	Standard Cox	1.15 (1.04, 1.28)	1.15 (0.98, 1.36)	1.15 (1.03, 1.28)	1.17 (0.96, 1.43)
	Women	Per SD	Standard Cox	1.07 (0.95, 1.20)	-	1.07 (0.94, 1.23)	-
Tumor subsite	Colon	Per SD	Standard Cox	1.16 (1.06, 1.26)	1.19 (1.05, 1.34)	1.17 (1.07, 1.27)	1.24 (1.07, 1.44)
	Colon (distal)	Per SD	Standard Cox	1.39 (1.12, 1.71)	-	1.32 (1.05, 1.66)	-

	Colon (proximal)	Per SD	Standard Cox	1.07 (0.95, 1.21)	1.02 (0.84, 1.24)	1.12 (1.00, 1.26)	1.22 (0.95, 1.56)
	Rectal	Per SD	Standard Cox	0.98 (0.83, 1.15)	-	0.95 (0.78, 1.16)	-
BMI	BMI < 25	Per SD	Standard Cox	1.16 (1.04, 1.30)	1.17 (0.95, 1.44)	1.17 (1.05, 1.31)	1.33 (1.02, 1.74)
	25 <= BMI <30	Per SD	Standard Cox	1.13 (0.98, 1.30)	-	1.16 (0.98, 1.37)	-
	BMI >= 30	Per SD	Standard Cox	0.93 (0.77, 1.12)	-	0.87 (0.70, 1.08)	-

HRs were estimated from Standard Cox or modified Cox, wherever applicable. The time function in all models was the time from CRC diagnosis to the event of death or the last follow-up. All models were stratified by country, and adjusted for sex, age at diagnosis (continuous), smoking status (never, past, current smoker), education (none, primary school, technical/professional school, secondary school, or longer education), sex-specific categories of physical activity (inactive, moderately inactive, moderately active, active), polyps of the large bowel (yes, no), alcohol consumption per day (continuous), intake per day of red meat, processed meat, fish and shellfish, calcium, dietary fiber, dairy products, vegetables and fruits (all variables were continuous), and adiposity measures including body mass index (BMI) (continuous), and residuals of waist circumference (WC) when regressed on BMI and height (ε(WC| BMI and height)). In the HR (95% CI) column, an en dash " - " indicates that the value is identical to the one on the left, as the excluded observation is not present in that group. The complete case analysis included 1046 participants (76% of the study sample). (*) A participant with an extreme FABP-4 level of 154.4 ng/mL—over twice the second-highest value (67.3 ng/mL)—was male, had a BMI below 25, and was diagnosed with proximal colon cancer. CS: cause-specific hazard; F&G: Fine-Gray model.

Supplementary Table 5. Estimates of the direct effect (DE) and indirect effect (IE) from total effect (TE) from mediation analyses of FABP-4 as a mediator in the association between pre-diagnosis BMI and mortality using the product-of-coefficients approach (the sensitivity mediation analysis).

	The total effect (TE) and 95% CI of BMI on the outcome	The direct effect (DE) and 95% CI of BMI on the outcome	The indirect effect (IE) and 95% CI of BMI on the outcome
Outcome: CRC-specific mortality	1.21 (1.10, 1.33)	1.13 (1.01, 1.26)	1.06 (1.01, 1.12)
Outcome: non-CRC-specific mortality	1.18 (1.05, 1.32)	1.12 (0.98, 1.27)	1.05 (1.00, 1.11)
Outcome: All-cause mortality	1.30 (1.06, 1.60)	1.19 (0.94, 1.50)	1.10 (0.99, 1.22)

In this analysis, we estimated the effect per 5 kg/m2 of BMI and per 1 SD of FABP-4. HRs for the mortality were estimated from a sub-distribution hazards Cox model as another approach for competing risk survival analysis (Fine-Gray model). HRs for all-cause mortality were estimated from conventional Cox proportional hazards models. The time function in all models was the time from CRC diagnosis to the event of death or the last follow-up. All models were stratified by country, and adjusted for sex, age at diagnosis (continuous), smoking status (never, past, current smoker), education (none, primary school, technical/professional school, secondary school, or longer education), sex-specific categories of physical activity (inactive, moderately inactive, moderately active, active), polyps of the large bowel (yes, no), alcohol consumption per day (continuous), intake per day of red meat, processed meat, fish and shellfish, calcium, dietary fiber, dairy products, vegetables and fruits (all variables were continuous).

Supplementary Table 6. The decompositions of the effect and mediation proportions mediated by FABP-4 in the relationship of **obesity (yes/no)*** and mortality using the causal approach.

	The total effect (TE) and 95% CI	The direct effect (DE) and 95% CI	The mediation proportion and 95%CI of the association mediated by FABP-4 (MP) (MP=1 - (DE/ TE))
Obesity* (yes/no) and the outcome			
Outcome: CRC-specific mortality	1.29 (1.03, 1.61)	1.17 (0.92, 1.48)	38.5% (8.3%, 81.2%) p = 0.005
Outcome: non-CRC-specific mortality	1.88 (1.28, 2.75)	1.64 (1.08, 2.48)	21.9% (5.5%, 57.4%) p = 0.03
Outcome: All-cause mortality	1.41 (1.17, 1.71)	1.27 (1.04, 1.55)	31.5% (12.6%, 59.5%) p = 0.0003

The mediation analysis was performed with the causal approach and assuming no interaction between FABP-4 and BMI, using SAS Macro ***%mediate,*** which was designed for time-to-event data with a stratified sample design. Mediation analyses were performed using the first imputed dataset. Other imputed datasets were similar and thus omitted. In this analysis, we estimated the effect per 5 kg/m2 of BMI and per 1 SD of FABP-4. The Cox proportional hazard model used in the mediation analysis has the time function in all models was the time from CRC diagnosis to the event of death or the last follow-up. All models were stratified by country, and adjusted for sex, age at diagnosis (continuous), smoking status (never, past, current smoker), education (none, primary school, technical/professional school, secondary school, or longer education), sex-specific categories of physical activity (inactive, moderately inactive, moderately active, active), polyps of the large bowel (yes, no), alcohol consumption per day (continuous), intake per day of red meat, processed meat, fish and shellfish, calcium, dietary fiber, dairy products, vegetables and fruits (all variables were continuous).

(*)BMI was categorized into obesity (BMI ≥ 30, n=253) and non-obesity (18.5 ≤ BMI < 30, n=1108). Ten participants (0.73%) with BMI < 18.5 were excluded.
